# Trade-off between benefits, harms and economic efficiency of low-dose CT lung cancer screening: a microsimulation analysis of nodule management strategies in a population-based setting

**DOI:** 10.1186/s12916-017-0924-3

**Published:** 2017-08-25

**Authors:** Marina Treskova, Ines Aumann, Heiko Golpon, Jens Vogel-Claussen, Tobias Welte, Alexander Kuhlmann

**Affiliations:** 1 Center for Health Economics Research Hannover (CHERH), Leibniz University of Hannover, Otto-Brenner-Str.1, 30159 Hannover, Germany; 2 Biomedical Research in Endstage and Obstructive Lung Disease Hannover (BREATH), Member of the German Center for Lung Research (DZL), Hannover, Germany; 3 Clinics for Pneumology, Hannover Medical School, Hannover, Germany; 4 Institute for Radiology, Hannover Medical School, Hannover, Germany

**Keywords:** LDCT lung screening, Lung cancer, NELSON, NLST, Nodule management protocol, Cost-effectiveness

## Abstract

**Background:**

In lung cancer screening, a nodule management protocol describes nodule assessment and thresholds for nodule size and growth rate to identify patients who require immediate diagnostic evaluation or additional imaging exams. The Netherlands-Leuvens Screening Trial and the National Lung Screening Trial used different selection criteria and nodule management protocols. Several modelling studies have reported variations in screening outcomes and cost-effectiveness across selection criteria and screening intervals; however, the effect of variations in the nodule management protocol remains uncertain. This study evaluated the effects of the eligibility criteria and nodule management protocols on the benefits, harms and cost-effectiveness of lung screening scenarios in a population-based setting in Germany.

**Methods:**

We developed a modular microsimulation model: a biological module simulated individual histories of lung cancer development from carcinogenesis onset to death; a screening module simulated patient selection, screening-detection, nodule management protocols, diagnostic evaluation and screening outcomes. Benefits included mortality reduction, life years gained and averted lung cancer deaths. Harms were costs, false positives and overdiagnosis. The comparator was no screening. The evaluated 76 screening scenarios included variations in selection criteria and thresholds for nodule size and growth rate.

**Results:**

Five years of annual screening resulted in a 9.7–12.8% lung cancer mortality reduction in the screened population. The efficient scenarios included volumetric assessment of nodule size, a threshold for a volume of 300 mm^3^ and a threshold for a volume doubling time of 400 days. Assessment of volume doubling time is essential for reducing overdiagnosis and false positives. Incremental cost-effectiveness ratios of the efficient scenarios were 16,754–23,847 euro per life year gained and 155,287–285,630 euro per averted lung cancer death.

**Conclusions:**

Lung cancer screening can be cost-effective in Germany. Along with the eligibility criteria, the nodule management protocol influences screening performance and cost-effectiveness. Definition of the thresholds for nodule size and nodule growth in the nodule management protocol should be considered in detail when defining optimal screening strategies.

**Electronic supplementary material:**

The online version of this article (doi:10.1186/s12916-017-0924-3) contains supplementary material, which is available to authorized users.

## Background

The National Lung Screening Trial (NLST) in the USA [1] has shown that lung screening with low-dose computed tomography (LDCT) can reduce lung cancer mortality by 20%, but it also can induce harms that the screened population may experience, i.e. false-positive findings, overdiagnosed cases, radiation-related deaths and interval cancers [2–5]. The largest lung screening trial in Europe, the Netherlands-Leuvens Screening Trial (NELSON) [6], used less stringent selection criteria and a different approach to patient management and has reported a reduced number of false positives compared to NLST [7]. The nodule management protocols of NLST and NELSON differ in applied measurement techniques (diametric vs volumetric assessment), follow-up algorithms and the definition of a cut-off nodule size indicating a cancer-positive result [8]. However, other differences between the studies (e.g. screened cohort, screening intervals) make it difficult to recognise the potential of nodule management approaches to succeed in the reduction of harms of screening.

Designing a screening program with an optimal balance between the benefits, harms and/or cost-effectiveness has become a major challenge for healthcare decision-makers who manage development of a lung screening program and decide on population selection strategies and screening intervals [7, 9, 10] as well as for clinicians who decide how to manage a screening-detected lung nodule [7].

Several modelling studies have examined trade-offs between the benefits and harms of LDCT screening and contributed to comprehension of the effects that eligibility criteria and screening intervals might have on its long-term screening performance and cost-effectiveness [11–18]. However, the effects of nodule management strategies have not been investigated in detail, and our understanding of how to proceed with a screening-detected nodule remains limited [19]. An algorithm for nodule assessment and management determines ways to prognosticate malignancy, defines core procedures of a screening program and may strongly influence the screening outcomes [20].

In this modelling study, we aimed to investigate the effects of the eligibility criteria and nodule management on the benefits, harms and cost-effectiveness of lung screening with LDCT in a population-based setting.

## Methods

### Microsimulation model

We developed a stochastic modular microsimulation model that simulated individual life histories focusing on the development of lung cancer and its progression from the onset of the first malignant cell to death from lung cancer.

**Fig. 1:**
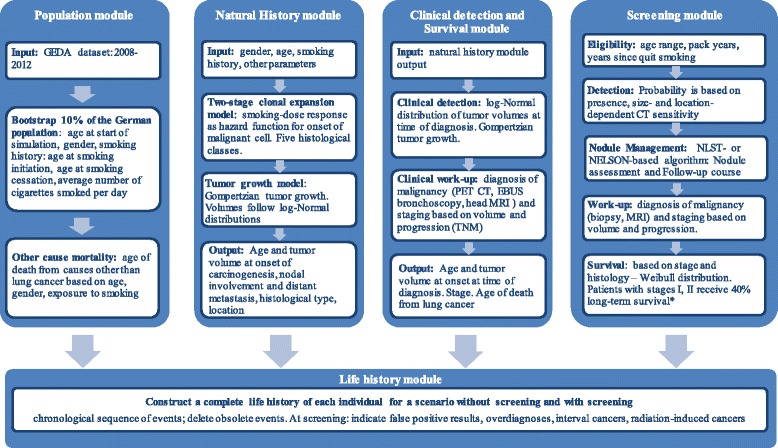
Structural modules of the microsimulation model. * refers to the case in which the patients would die from lung cancer in the no screening scenario

The model consists of the following structural modules: population, natural history, clinical detection, survival, screening and life history (Fig. 1). The model was populated with 10% of the German population aged 40 years and older. Data on smoking behaviour was obtained from the German Health Update (GEDA) survey (years 2009–2012) [21], and the demographic structure of 2012 was obtained from the German statistical office [22].

The natural history module contains a biological two-stage clonal expansion (TSCE) model [23] and a tumour growth component and simulates a complete flow of events in the development of lung cancer (details are available in Additional file 1: Section 1.1.2). The TSCE model defines the individual age at the onset of the first malignant cell and the histologic cancer type: small cell, large cell, squamous cell carcinoma, adenocarcinoma or adenocarcinoma in situ (AIS). The progression of lung cancer is described via tumour growth, lymph nodes involvement and metastasis. The tumour growth is defined by a Gompertz function [24] (Additional file 1: Section 1.1.4.2). This function describes the relation between time and the tumour volume. For a specific tumour volume the function gives the time needed to reach this volume and vice versa. The module uses the age at the onset of the first malignant cell and the time to reach the stage-specific volume and gives the age at different stages of the progression of the disease. Threshold tumour volumes at the stages of nodal involvement, distant metastases and clinical diagnosis are randomly drawn from log-normal distributions (Additional file 1: Table S5), and the tumour growth model is applied to calculate the corresponding ages of the individual. The clinical detection module determines the stage of lung cancer (I, II, III, IV) according to the tumour-node-metastasis (TNM) staging system based on the tumour volume and spread (local, nodal involvement, distant metastasis) at the age of diagnosis (Additional file 1: Section 1.1.3).

The lung cancer survival is modelled as long-term survival, which lets the individual live until death from other causes, and short-term survival in years, which follows the Weibull distribution [25]. The parameters vary over the histological classes and stages at the time of diagnosis (Additional file 1: Table S1, Section 1.1.3) [25].

The screening module (Additional file 1: Section 1.1.5) contains several structural components: eligibility assessment, screening-detection, nodule management (including follow-up), diagnostic work-up and lung cancer survival. For each individual it creates a screening schedule based on eligibility criteria and nodule management protocol. At each screening exam, the module checks for presence of a lung nodule and determines its volume using the tumour growth function, the individual’s age and the age at the onset of the first malignant cell. Screening-detection depends on the location and volume of the tumour and the sensitivity of the CT scan (Additional file 1: Section 1.1.5.2, Table S7). Individuals with a detected nodule proceed with a nodule management algorithm. The nodule management algorithm defines the threshold values of the nodule size and tumour growth and indicates the patients who require immediate diagnostic work-up or undergo additional imaging exams (follow-up course).

**Fig. 2:**
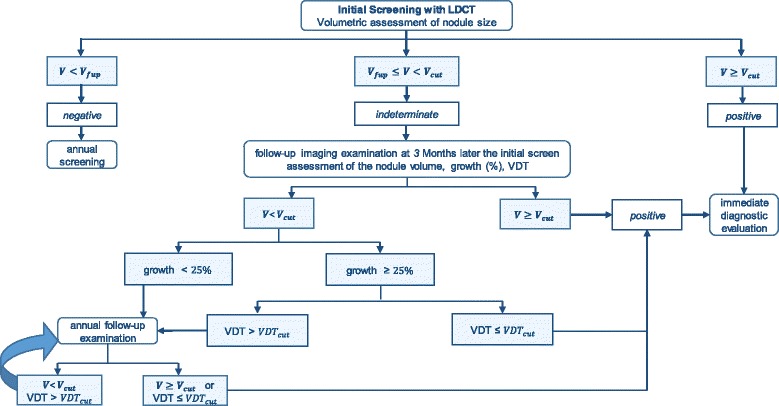
Schematic representation of modelled NELSON-like nodule management protocol. *V*_cut_ and *VDT*_cut_ indicate the threshold values of the volume and volume doubling time which indicate a cancer positive result. *V*_fup_ represents low threshold volume for a follow-up examination

**Fig. 3:**
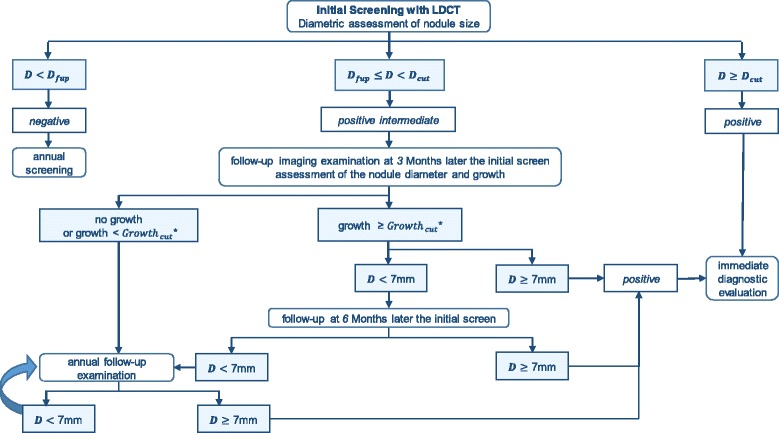
Schematic representation of modelled NLST-like nodule management protocol. *Growth is calculated as a percentage increase in the diameter vs the diameter at the initial screening. *D*_cut_ and *Growth*_cut_ indicate the threshold values of the diameter and percentage increase in the diameter that indicate a cancer positive result. *D*_fup_ represents low threshold diameter for a follow-up examination

Two nodule management algorithms were designed based on those used in the NELSON and NLST trials. Schematic representations of the algorithms are given in Figs. 2 and 3. The modelled NELSON-like nodule management algorithm includes volumetric assessment of the nodule size. Based on the nodule volume, the patients undergo either the next screening round (if negative), a follow-up exam 3 months later (if indeterminate) or an immediate diagnostic evaluation (if positive) [6, 26]. At the follow-up examination, tumour growth and tumour volume doubling time (VDT [6]) are assessed as an additional malignancy predictor.

The NLST-like nodule management algorithm includes diametric assessment of the nodule size and defines three categories of screening results: negative, positive intermediate and positive (Fig. 3). In contrast to the NELSON-like nodule management algorithm, individuals with positive intermediate initial results undergo a course of follow-up chest imaging exams where tumour growth is assessed as a change (%) in the nodule diameter relative to the result at the initial screening. The follow-up can occur with a fixed periodicity: at 3, 6 and 12 months after the initial screening. Additional details on the modelled nodule management protocols are available in Additional file 1: Section 1.1.5.3. Individuals with lung cancer, defined in the management algorithm, undergo the diagnostic work-up component (Additional file 1: Section 1.1.5.4), and the tumour is staged according to TNM classification based on the volume and spread. These patients are withdrawn from the regular screening schedule.

**Fig. 4:**
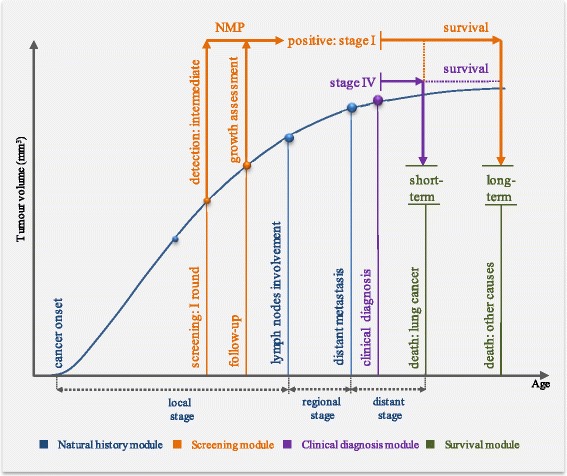
Schematic representation of modelled tumour growth and interaction between the natural history, screening, clinical diagnosis and survival modules. *NMP* nodule management protocol. The curve schematically represents the tumour growth. Figure does not reflect the scales. The natural history module contains a biological two-stage clonal expansion (*TSCE*) model and a tumour growth component and simulates for each individual the age at the onset of carcinogenesis, its histological features, the age and tumour size at the lymph nodes involvement and distant metastasis. The TSCE model simulates age at the cancer onset for each histological class. The final histological class for the individual is determined based on the competing risk (the lowest age at onset). The tumour growth component applies a Gompertz function which describes the relation between time (age) and the tumour volume. The clinical diagnosis model determines the age at lung cancer diagnosis and stage of the tumour according to TNM classification using the tumour growth model and information on the tumour progression from the natural history module. The screening module simulates an individual screening schedule based on the eligibility criteria. It applies the tumour growth module to determine the tumour volume at age of screening and uses information on the tumour progression for staging the screen-detected tumour according to TNM classification. The survival model determines the age of death based on the tumour stage and histological class. The figure illustrates a case where an individual in the no screening scenario develops a lung cancer tumour and is eventually symptomatically diagnosed with lung cancer at stage IV. The patient dies from lung cancer in the no screening scenario. In the screening scenario, a nodule (tumour) is detected in the first round of screening. The screen-detected nodule is small for the patient to undergo an immediate diagnostic evaluation. The patient undergoes a follow-up exam, where the growth is assessed according to the NMP. The growth and/or the volume doubling time meet the definition of cancer according to the NMP. The screen-detected tumour is at the local stage, and the patient is diagnosed with lung cancer at stage I in the screening scenario. The patient is cured and dies from other causes. The model calculates life years gained for each individual in the screened cohort

We assume that individuals with screen-detected lung cancer live at least as long as they would in the no screening scenario. In the screening module lung cancer, survival component alters the age of death from lung cancer for the persons with a screen-detected lung cancer at stages I and II: if they die from lung cancer in the no screening scenario, they receive 40% probability of long-term survival [25]. The screening module sums up imaging exams, work-ups, complications and treatments. The life history module computes false positives and interval cancers and calculates overdiagnosed cases and deaths from radiation-induced cancer (Additional file 1: Section 1.1.6). Figure 4 gives a schematic representation of the modelling of the tumour growth and interaction between the natural history, screening, clinical diagnosis and survival modules.

### Screening scenarios

At base case, a 5-year LDCT annual lung screening program with perfect adherence was evaluated. Overall, 76 scenarios were constructed using variations of the eligibility criteria (four different screened populations) and nodule management protocol (defined as the NELSON-like or NLST-like protocol) (Table 1). The outcomes were projected over the course of a lifetime. Lung cancer-specific mortality reduction and false-positive cases were calculated for the screened cohort.

**Table 1:** Characteristics of the evaluated screening scenarios

Characteristics	Considered variations
*Eligibility criteria*	
Population:	50-74-30-15
Values arranged as	(eligibility criteria of the NLST clinical trial)
age at begin smoking - age at quit smoking - minimum pack years - maximum years since quitting smoking	55-80-30-15
	(as recommended by the US Preventive Services Task Force (USPSTF) for lung screening with LDCT [2])
	50-75-15-9
	(less restrictive eligibility criteria, similar to the NELSON trial)
	55-75-40-10
	(more restrictive eligibility criteria) [18]
*Nodule management algorithm*	
NELSON-like	*VDT*_cut_ = 400 days – *V*_cut_ = 500 mm^3^
Scenario is characterised by the threshold value of the volume doubling time (*VDT*_cut_) and the cut-off volume (*V*_cut_) for cancer positive	(values of the NELSON clinical trial)
	*VDT*_cut_ = 400 days – *VDT*_cut_ = 300 mm^3^
	*VDT*_cut_ = 400 days – *VDT*_cut_ = 400 mm^3^
	*VDT*_cut_ = 400 days – *VDT*_cut_ = 750 mm^3^
	*VDT*_cut_ = 300 days – *VDT*_cut_ = 500 mm^3^
	*VDT*_cut_ = 600 days – *VDT*_cut_ = 500 mm^3^
	*VDT*_cut_ = 300 days – omitting *VDT*_cut_^a^
	*VDT*_cut_ = 400 days – omitting *VDT*_cut_^a^
	*VDT*_cut_ = 600 days – omitting *VDT*_cut_^a^
	*V*_fup_ = 80 mm^3^ – *VDT*_cut_ = 400 days – *VDT*_cut_ = 500 mm^3^ ^b^
NLST-like	*Growth*_cut_ = 10% – *D*_cut_ = 10 mm
Scenario is characterised by the threshold value of the tumour growth and the diameter (*D*_cut_) for cancer positive	(values of the NLST clinical trial)
	*Growth*_cut_ = 10% – *D*_cut_ = 9 mm
	*Growth*_cut_ = 10% – *D*_cut_ = 11 mm
	*Growth*_cut_ = 7.5% – *D*_cut_ = 10 mm
Tumour growth (threshold growth, *Growth*_cut_) is defined as a percentage increase in diameter	*Growth*_cut_ = 12.5% – *D*_cut_ = 10 mm
	*Growth*_cut_ = 7.5% – omitting *D*_cut_^a^
	*Growth*_cut_ = 10% – omitting *D*_cut_^a^
	*Growth*_cut_ = 12.5% – omitting *D*_cut_^a^
	*D*_*f*up_ = 5 mm – Growth = 10% – *D*_cut_ = 10 mm^b^

^a^In these scenarios nodule growth is taken as a single malignancy predictor

^b^In these scenarios a higher nodule size for the follow-up exams (*V*_fup_; *D*_fup_) is used according to the British Thoracic Society guidelines [29]. In other scenarios the value of nodule size for follow-up exams is applied according to the trials as 4 mm (NLST-like) and 50 mm^3^ (NELSON-like)

### Health economics

Costs included LDCT exams, staging tests and lifetime treatment (Additional file 1: Table S8). Expenditures of lifetime treatment, due to limitations of available cost data for Germany, were calculated via application of cost variations across cancer stages obtained from the UK cost data [27] compared with the German cost data [28] (Additional file 1: Section 1.3). The lifetime treatment costs for patients with early-stage and advanced cancers were 45,803 euro for stages I/II and 30,101 euro for stages III/IV.

Cost-effectiveness was represented by average and incremental cost-effectiveness ratios (ACER and ICER, respectively). Life years gained (LYG) and averted lung cancer deaths constituted the main benefits of the screening. We applied equal (3%) and differential (3% for costs and 1.5% for LYG) annual discounting. A health insurance perspective was used.

### Sensitivity analyses

One-way sensitivity analyses were performed to assess variations of the cost-effectiveness after altering assumptions about LDCT sensitivity parameters, parameters of long-term survival after screening, attendance rate cost per CT exam and lifetime treatment costs (Additional file 1: Section 1.4).

## Results

### Benefits and harms of screening

Annual screening led to a 9.7–12.8% reduction in lung cancer mortality in the screened cohorts. Relative to usual care, where 79% of cancers were diagnosed at stages III and IV, a screening program shifted the majority of diagnoses towards the early-staged cancers (stages I/II accounted for 66.4–71.7%). Adenocarcinomas (around 50%), squamous cell carcinomas (around 23.4%) and AIS (around 18.2%) constituted the majority of screening-detected cancers. Around 77.2% of screening-diagnosed AIS were overdiagnosed cases. Overdiagnoses constituted 9–21.5% of all screening-detected lung cancers. Small cell carcinomas were rarely detected at screening (around 5.35%) but constituted 56% of all interval cancers. False-positive diagnoses constituted 59.4–96% of all screening findings.

Eligibility criteria have a considerable influence on the main outcomes of a large-scale screening program. Evaluated scenarios with a selection of people similar to the eligibility criteria of the NLST clinical trial (55-74-30-15) gained 192,147–240,626 life years (ranged over the variations of the nodule management protocol) and 20,335–25,467 averted deaths due to lung cancer and induced 3780–5069 million euro costs additional to no screening. The scenarios with the increased threshold of exposure to smoking (40 pack-years and maximum 10 years since quitting) limited the screened population and yielded around 31% less LYG (133,222–164,864) and 29% less averted deaths (14,373–17,889) and induced 37% fewer costs (2231–3178 million euro). Increasing the stopping age to 80 (55-80-30-15) yielded around 8.3% additional LYGs (207,468–260,807) and 14.4% more averted lung cancer deaths (23,029–29,165) compared to the 55-74-30-15 scenarios and a slightly increased reduction in lung cancer mortality (12.4%); however, it induced around 13.5% more costs (4159–5811 million euro) and the highest rate of overdiagnosis. Scenarios with the least restrictive eligibility criteria, 50-75-15-9, resulted in 50.5% more LYGs (295,093–362,039) and 45.6% more averted lung cancer deaths (30,147–37,075) vs the 55-74-30-15 scenarios; however, they led to 57% more healthcare costs (6447–8026 million euro additional to no screening) and a higher number of CT scans.

Generally, the scenarios with the NELSON-like nodule management protocol resulted in 1.1–1.3% fewer lung cancer findings, but around 2.3% more findings of cancer at an early stage, 3.3–3.4% more cases of averted lung cancer deaths, around 3% more LYGs, 0.1–0.9% fewer overdiagnosed cases and around 3% more interval cancers than the NLST-like strategies. The NLST-like scenarios yielded around 51–57.4% more follow-ups of malignant nodules and considerable additional costs.

Overall, across the evaluated 76 scenarios, a few tendencies in the effects of the nodule management protocols could be seen: (1) increasing the threshold for nodule size for a cancer-positive diagnosis slightly decreased overdiagnosis, LYG and averted lung cancer deaths; (2) decreasing the cut-off size yielded more overdiagnosed cases but did not improve numbers of LYG and averted lung cancer deaths; (3) altering threshold values for a cancer indicating nodule growth when the cut-off volume stayed the same did not appreciably change the rates of LYG and averted lung cancer deaths; (4) application of VDT or an increase in diameter as a single malignancy predictor in the two-step framework remarkably reduced the accuracy of lung cancer diagnosis, but it also notably decreased rates of overdiagnosis. Increasing the threshold nodule size for a follow-up in the NELSON-like scenarios from a volume of 50 mm^3^ to 80 mm^3^ and in the NLST-like scenarios from a diameter of 4 mm to 5 mm, as recommended by the British Thoracic Society [29], led to a 5% and 4% decrease in overdiagnosis and a 3.7–5% decline in LYG and averted lung cancer deaths.

### Cost-effectiveness of screening

**Fig. 5:**
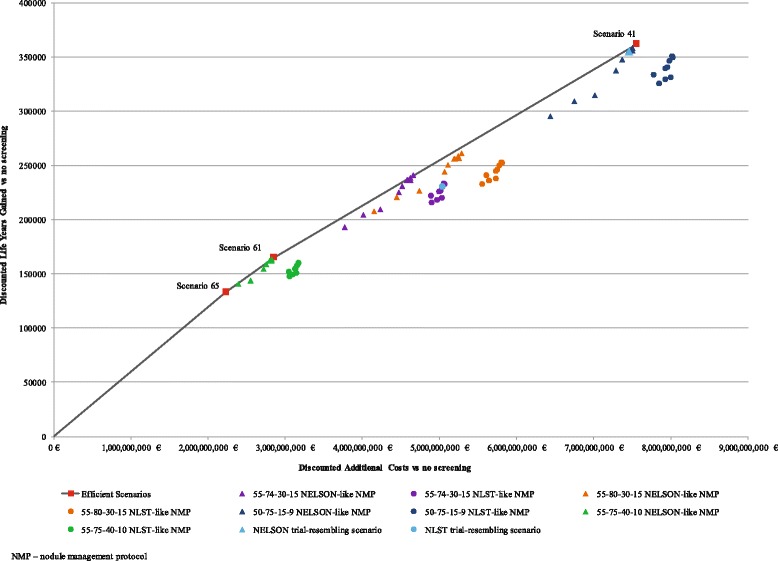
The cost-effectiveness (cost per life year gained) of all evaluated scenarios and the scenarios that constitute an efficient frontier. The figure illustrates three scenarios judged to be efficient based on the cost per life year gained ratio. These scenarios constitute the efficient frontier. The other 73 evaluated scenarios are illustrated according to the population selection criteria and applied nodule management protocol (*NMP*). The figure illustrates the four evaluated eligibility criteria in different colours: 55-75-40-10 is given in *green*, 55-74-30-15 is given in *violet*, 55-80-30-15 is given in *orange* and 50-75-15-9 is given in *dark blue*. The scenarios which apply the NLST-like nodule management are illustrated with a *circular shape*. The scenarios which apply the NELSON-like nodule management are illustrated with a *triangular shape*. The figure does not specify in colour or a shape the evaluated variations of the threshold values for the tumour size and growth in the NELSON-like and NLST-like scenarios. The scenarios which resemble the eligibility criteria and nodule management protocols of the NLST and NELSON clinical trials are illustrated in *light blue*. Descriptions of the scenarios are given in Table 1. Main outcomes and cost-effectiveness of the 76 baseline screening scenarios are given in Additional file 1: Table S12

**Fig. 6:**
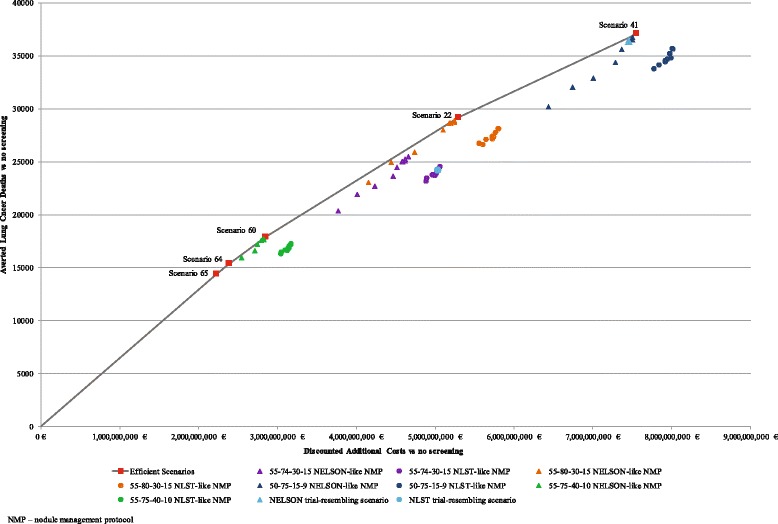
The cost-effectiveness (cost per averted lung cancer death) of all evaluated scenarios and the scenarios that constitute an efficient frontier. The figure illustrates five scenarios judged to be efficient based on the cost per averted lung cancer death ratio. These scenarios constitute the efficient frontier. The other 71 evaluated scenarios are illustrated according to the population selection criteria and applied nodule management protocol (*NMP*). The figure illustrates the four evaluated eligibility criteria in different colours: 55-75-40-10 is given in *green*, 55-74-30-15 is given in *violet*, 55-80-30-15 is given in *orange* and 50-75-15-9 is given in *dark blue*. The scenarios which apply the NLST-like nodule management are illustrated with a *circular shape*. The scenarios which apply the NELSON-like nodule management are illustrated with a *triangular shape*. The figure does not specify in colour or a shape the evaluated variations of the threshold values for the tumour size and growth in the NELSON-like and NLST-like scenarios. The scenarios which resemble the eligibility criteria and nodule management protocols of the NLST and NELSON clinical trials are illustrated in *light blue*. Descriptions of the scenarios are given in Table 1. Main outcomes and cost-effectiveness of the 76 baseline screening scenarios are given in Additional file 1: Table S12

ACER ranged from 16,754 to 24,160 euro/LYG (Fig. 5) and from 155,287 to 230,678 euro/averted lung cancer death (Fig. 6). Out of the 76 evaluated scenarios, three scenarios were judged to be efficient based on their cost/LYG ratio and five scenarios based on their cost per averted lung cancer death ratio (Table 2).

**Table 2:** Main outcomes of the efficient scenarios

Scenario	Scenario characteristics^a^	Detected cancers at an early stage (I/II), %	Reduction in lung cancer mortality, %	Lung cancer deaths averted	Discounted life years gained	Interval cancer cases	Overdiagnosed cases	Overdiagnosis, %	Discounted total cost, million euro	Discounted additional costs vs no screening, million euro	Cost per life years gained vs no screening (uniform discounting), euro	Discounted cost per lung cancer death averted vs no screening, euro	ICER vs the previous efficient scenario, euro
Efficient scenarios based on the cost per life year gained ratio													
Scenario 65	55-75-40-10-NELSON-VDT300-none	67.31	9.95	14,373	133,222	23,057	6733	9.48	10,892	2232	16,754	155,287	16,754
Scenario 60	55-75-40-10-NELSON-VDT400-V300	71.35	12.38	17,889	164,864	19,854	17,892	9.69	11,516	2855	17,321	159,625	19,707
Scenario 41	50-75-15-9-NELSON-VDT400-V300	72.39	11.90	37,075	362,039	43,331	32,183	17.78	29,456	7556	20,870	203,792	23,804
Efficient scenarios based on the cost per averted lung cancer death ratio													
Scenario 65	55-75-40-10-NELSON-VDT300-none	67.31	9.95	14,373	133,222	23,057	6733	9.48	10,892	2232	16,754	155,287	155,287
Scenario 64	55-75-40-10-NELSON-VDT400-none	67.95	10.65	15,395	140,490	21,367	9184	11.73	11,057	2397	17,059	155,675	161,124
Scenario 60	55-75-40-10-NELSON-VDT400-V300	71.35	12.38	17,889	164,864	19,854	17,892	19.69	11,516	2855	17,321	159,625	184,009
Scenario 22	55-80-30-15-NELSON-VDT400-V300	70.95	12.80	29,165	260,807	32,071	33,473	21.76	18,846	5296	20,307	181,597	216,454
Scenario 41	50-75-15-9-NELSON-VDT400-V300	72.39	11.90	37,075	362,039	43,331	32,183	17.78	29,456	7556	20,870	203,792	285,630

^a^Scenarios are named ranging these values as follows: ”population selection criteria-nodule management protocol-threshold values for growth rate and nodule size”

The scenarios that featured NELSON and NLST clinical trials were less efficient (Figs. 4 and 5, NLST- and NELSON-resembling scenarios). Compared to the NELSON-like scenarios, NLST-based screening over 5 years of annual screening would result in considerably more total costs (around 450 million euro) while yielding around 800 fewer averted deaths. Considering the cost per life year gained ratio, characteristics of the not-dominated scenario included the most restrictive eligibility criteria (55-74-40-10) and the NELSON-like nodule management protocol which applies the assessment of VDT 3 months after the initial screening as a sole malignancy predictor (Scenario 65, Table 2). The scenario yielded an ICER of 16,754 euro/LYG. The second efficient scenario (Scenario 60, Table 2) combined the threshold VDT of 400 days and a cut-off nodule volume of 300 mm^3^. This scenario gained 31,642 additional life years for an incremental cost of 19,707 euro/LYG. The third efficient scenario (Scenario 41, Table 2) applied the same nodule management protocol and less stringent eligibility criteria (50-75-15-9). It gained an additional 197,174 life years for an incremental cost of 23,837 euro/LYG.

Two of the five scenarios, which were judged to be efficient based on averted cancer deaths, included the most restrictive selection criteria (55-75-40-10) and assessment of VDT 3 months later than the initial screening as a sole malignancy predictor for individuals with initial findings over 50 mm^3^ in volume (Scenarios 64 and 65, Table 2). The scenario with the lowest ICER of 155,287 euro per averted death (Scenario 65 with threshold VTD of 300 days) yielded 14,373 averted deaths. Increasing the threshold value of VDT to 400 days gained an additional 1000 averted deaths for an incremental cost of 161,124 euro (Scenario 64). The inclusion of the cut-off volume of 300 mm^3^ (Scenario 60) into the nodule management algorithm yielded 2500 more averted deaths for an incremental cost of 184,009 euro. The scenario with less restrictive selection criteria of exposure to smoking and the increased stopping age (55-80-30-15, Scenario 22) yielded 11,276 more averted lung cancer deaths for an ICER of 216,454 vs the previous efficient scenario. The scenario with the largest number of LYG (Scenario 41) is also the scenario with the largest yield of averted cancer deaths (37,075) for an ICER of 285,630 euro per averted death.

### Sensitivity analyses

**Fig. 7:**
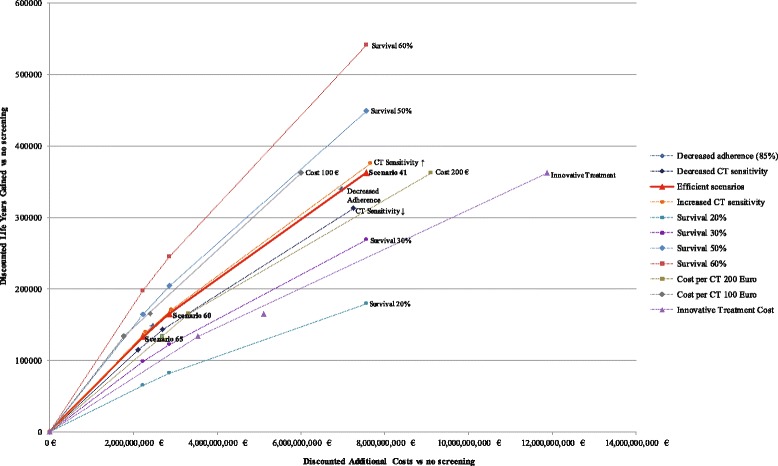
The cost-effectiveness of the efficient scenarios (cost/LYG) in the sensitivity analyses. Scenario 60 is not efficient under conditions of decreased adherence, innovative treatment and cost per CT exam of 100 euro. Scenario 65 is not efficient when cost per CT exam is 200 euro

Figure 7 illustrates the discounted life years and additional costs (vs no screening) for the three efficient scenarios (Scenarios 65, 60 and 41) and for their variations in the sensitivity analyses. The main cost-effectiveness drivers are cost per CT exam, treatment costs and lung cancer long-term survival probability in screening. Relative to the baseline long-term survival probability (40%), its reduction to 20% led to a more than 50% reduction in LYG and averted deaths with a more than 100% increase in cost/LYG. Increase in cost per CT exam would have a stronger adverse effect on the cost-effectiveness if the less restrictive eligibility criteria were used. More expensive treatment with innovative targeted medication at a lifetime cost of 77,702 euro [28] would increase the ACER by 65%. An increase of the CT sensitivity for smaller nodules would slightly improve the cost-effectiveness; a 20% decrease of the sensitivity would lead to a more than 10% increase in ACER. Compared with perfect adherence (100%), decreasing adherence to 85% for the years following the initial screening led to a modest decline of ACER (around 1%). Screening strategy 60 (55-75-40-10-NELSON-VDT400-V300) becomes inefficient in the scenarios of the decreased adherence, decreased cost per CT exam and increased treatment cost (i.e. innovative treatment scenario). Due to high rates of detection and overdiagnosis, factors that increase ratio of treatment relative to the costs of screening have adverse effects on ICER compared to the previous efficient scenario. Scenario 65 (55-75-40-10-NELSON-VDT300-only) in turn becomes inefficient under conditions of increased screening costs. Expanding the period of the screening to 10 years did not considerably influence the cost-effectiveness. Detailed results of baseline and sensitivity analyses are available in Additional file 1: Sections 2.2–2.4.

## Discussion

The microsimulation analysis shows that lung cancer screening of the high-risk population in Germany can be cost-effective. A program with less restrictive eligibility criteria would be more effective but would induce a higher ICER due to screening of people with a lower risk of lung cancer development. The selection of heavier smokers and limitation of the screened population yields the lowest cost-effectiveness ratio compared with the no screening scenario. Increasing the stopping age, i.e. following the recommendations of the USPSTF [2], prevents additional deaths from lung cancer but also leads to considerable additional costs and increased overdiagnosis. Overall, if decision-making is based on the results of estimation of efficiency of an intervention, the scenarios that constitute an efficient frontier should be considered for implementation because other scenarios are proven to be less efficient. The efficiency frontier itself is shaped by the applied measure of efficiency.

Our study provides two efficiency frontiers based on weighting two major benefits of screening (LYG and averted lung cancer deaths) against the resulting costs (additional costs relative to the no screening scenario). The comparative modelling study by de Koning et al. for the USPSTF [2] in turn used “screenings per lung cancer death averted” as a measure of the efficiency. Based on this measure, the authors concluded that the eligibility criteria 55-80-30-15 are more efficient than the original NLST criteria. Considering their results for the screenings per LYG as the efficiency measure, the 55-80-30-15 scenario is still more effective but less efficient than the 55-75-30-15 scenario. Although we applied the additional cost in the numerator of the cost-effectiveness ratio, our findings are consistent with those by de Koning et al.: the selection criteria 55-80-30-15 are more efficient than 55-74-30-15 if the efficiency is estimated using the cost per averted lung cancer death ratio but less efficient based on the cost per life year gained ratio. Overall, both efficient frontiers outlined in our study do not include scenarios with the 55-74-30-15 population selection criteria.

Additionally, the efficiency measure “screenings per lung cancer death averted” used by the USPSTF to give the recommendation for screening does not include the harms of overdiagnosis. In our study, the cost side of the cost-effectiveness ratio includes the costs (related to screening and treatment) of the overdiagnosed cases and by that the overdiagnosis is incorporated into the measure of efficiency. In screening for lung cancer, overdiagnosis is thought to be “the most extreme form of length-time bias” [30]. These cases include patients with tumours that would never have caused symptoms or been diagnosed in clinical settings. Stopping screening at age 80 leads to substantially higher numbers of overdiagnosed cases. This also has been shown in the studies by ten Haaf et al. [18] and de Koning et al. [2]. In our analysis, the costs of the overdiagnosed cases account for about 30% of the difference in costs between the 55-80-30-15 and 55-74-30-15 scenarios. If the impact of overdiagnosis on the quality of life was included into estimation of the efficiency, it would be expected to further reduce the effectiveness and efficiency of the screening of an older population.

Ideally, all benefits and harms which are considered to be relevant for the identification of an optimal screening strategy should be included into the measure of effectiveness and efficiency. However, this would require a defined efficiency threshold [31] or prioritisation and weighting of the benefits and harms made by decision-makers. The aim of our study was to evaluate strategies for an introduction of a large-scale lung screening in Germany and to outline and discuss efficient scenarios rather than provide a solid recommendation.

Patient management strategy has a strong influence on long-term performance and cost-effectiveness of lung screening. The NELSON- and NLST-like nodule management protocols are comparably effective in reducing lung cancer mortality; however, the NELSON-like strategy is more successful in detection of early-stage lung cancers, yields fewer follow-up exams, and saves costs, and therefore may be preferable in clinical practice. The efficient screening scenarios highlight the combinations of the eligibility criteria and NELSON-like nodule management protocols, which may yield additional benefits without increasing the ICER. The cut-off volume for immediate diagnostic evaluation is a key element of the nodule management strategy. In the NELSON trial, the cut-off volume was defined as 500 mm^3^ and higher. Our results show that a decrease to 300 mm^3^ would be a more cost-effective strategy and may be justified for clinical practice. From the health economics perspective, our findings support previous inferences made by Horeweg and colleagues, who assessed probabilities of cancer development based on the NELSON data and concluded that patients with a screen-detected nodule of volume of 300 mm^3^ or more should undergo immediate diagnostic work-up [20]. Additionally, variations of the nodule management elements show that the assessment of VDT at follow-up becomes more important if the threshold nodule volume is less restrictive (more than 500 mm^3^).

It is important to note that a decreased cut-off volume may lead to an increased number of overdiagnosed cases. In our analysis, the majority of the overdiagnosed cases were patients with slowly growing adenocarcinomas and AIS. Due to their slow growth, these are rarely symptomatically diagnosed [32] but may be detected by screening. Our results also suggest that scenarios which exclude the threshold nodule volume as the indicator for immediate diagnostic evaluation and apply the assessment of VDT (at the follow-up exam 3 months later) as a sole malignancy predictor can considerably reduce overdiagnosis; however, this can come at a high price of missing LYG and averted lung cancer deaths. Although a number of cases and the costs of overdiagnosis can be calculated in model settings, their effects on the health outcomes and quality of life need to be further investigated and quantified.

In this study, the effect of screening on quality of life could not be included in the analysis due to lack of German data on values of quality-adjusted life years (QALYs) across the lung cancer stages, sexes and age groups. The cost-effectiveness ratios in terms of costs per QALY gained would be expected to be notably higher than the estimated cost/LYG ratios [7]. As long as the screening shifts the major part of diagnoses towards the early-stage cancers, more patients are likely to receive a resection operation. These patients are reported to have a considerably impaired quality of life during the first 2 years after lung resection but it may improve later [33, 34]. Additionally, with the application of QALYs, the negative effects of overdiagnosis and false positives would considerably increase [35, 36].

Several limitations are worth noting. Tumour growth is simulated using a Gompertzian growth model which does not capture abrupt changes in the development (growth) of the tumour. However, other studies have shown that cancer tumour growth can be well approximated by a Gompertz function [37]. Our approach to modelling of false positives is rather simplified and does not allow for a comprehensive analysis of the false-positive outcomes at screening. The question of how to decrease the number of false-positive cases remains unanswered and requires additional information, which clinical trials may provide in the future. The limitations form a direction for further research into lung screening in Germany. There is a need to collect detailed data on tumours at time of diagnosis about their size, stage, smoking habits of the patients, treatment costs and quality of life of German patients with lung cancer, along with factors affecting screening uptake among the target groups. The cost-effectiveness of a combination of a smoking cessation intervention with a screening program is worth investigating.

Despite the limitations, the present study contributes insights on the impacts of the nodule management protocol on the effectiveness and cost-effectiveness of the introduction of a large-scale lung screening program. Additionally, this work is the first modelling study of microsimulation design which examines cost-effectiveness of the introduction of a large-scale lung screening program in a European country. The presented findings are comparable to the results reported in NLST [38] and previous modelling studies [2, 14, 18]. Because the developed model contains very little input obtained from the NLST data, comparison with the outcomes of NLST may serve as validation of the model. Outcomes of the model for the scenario most similar to NLST (Additional file 1: Section 2.2, Table S10) show a resembling distribution of histologic classes and stages of lung cancers detected at screening and interval cancers [1]. NLST reports lung cancer mortality outcomes relative to radiography for a median follow-up of 6.5 years [38]. We could compare these with the outcomes of our microsimulation model for 7 years of follow-up (Additional file 1: Table S14). In our analysis lung cancer mortality is considerably higher in the no screening settings and in the NLST-resembling scenario than reported in the trial; however, the differences between mortality rates in the screening and no screening scenarios are close to the differences in mortality rates between LDCT screening and radiography observed in NLST. Considering lung cancer mortality reduction as a ratio, due to the higher mortality rates the model reports a lower percentage (around 16%) in comparison to NLST (21%) [38]. However, it still falls into the range given by confidence interval calculations in NLST. Our model also predicts a higher all-cause mortality rate than observed in NLST. The higher mortality may be caused by an older population and a larger proportion of current smokers in the screened cohorts; additionally, the settings of a clinical study and a possible “healthy-volunteer” effect [38] may positively affect the mortality outcomes of the clinical trial.

Overall, reduction in lung cancer mortality calculated over a lifetime course ranges from 9.7 to 12.8% and stays similar to the values reported in previous studies [2, 18]. Furthermore, the difference in the mortality rates between the no screening and screening arms is the basis for the economic evaluation, and it is very similar to that of the NLST trial. We therefore suggest that application of survival data other than that reported in NLST has a limited influence on the presented findings.

The cost-effectiveness has been analysed in modelling studies in the USA [14] and Canada [18]. Due to the application of QALYs in the study by McMahon et al. (USA), the obtained cost-effectiveness ratios cannot be compared. Comparing the findings to the study by the Canadian team, the cost-effectiveness ratios obtained in this study lie within the range given by ten Haaf et al., despite differences in the applied cost inputs [18].

Overall, applicability of the presented results for other countries depends on the similarity of the cost structure and heavy smoker prevalence between the countries. The sensitivity analyses show that cost-effectiveness ratio is driven by cost per CT exam and other screening-related costs. The cost per CT exam taken in our study is not Germany-specific and is varied in the sensitivity analyses, giving an opportunity to transfer the results to other countries. The costs of lung cancer treatment tend to be higher in Germany, as in other European countries [39]. Pharmaceutical companies continue to keep prices higher in Germany based on the expectation that German prices will become reference prices for the pharmaceuticals in other European countries [40]. Lower costs of screening and lung cancer treatment in other countries may decrease the cost-effectiveness ratio and make population-based lung screening more cost-effective.

Due to differences in exposure to smoking in populations, cost structures and approaches to lung cancer treatment, efficient scenarios of lung screening can vary between the European countries. However, the main findings on the impacts of the nodule management protocol and population selection criteria on the cost-effectiveness of the screening can be applied to other countries.

## Conclusions

This study quantifies the effect of nodule management approaches on the benefits, harms and cost-effectiveness of lung screening. Our analysis shows that the nodule management protocol has a considerable effect on screening performance and should be considered in greater detail when defining optimal screening strategies. It is the first cost-effectiveness analysis of lung cancer screening using a microsimulation design performed in a population-based setting in Germany. These results can support decision-making processes in lung cancer prevention and direct creation of guidelines for LDCT lung cancer screening to benefit the German population.

## Additional file

Additional file 1: Microsimulation model components, modelling methods, input parameters and additional summaries of results. (PDF 3708 kb)

## Abbreviations

ACER: Average cost-effectiveness ratio
AIS: Adenocarcinoma in situ
ICER: Incremental cost-effectiveness ratio
LDCT: Low-dose computed tomography
LYG: Life years gained
NELSON: Netherlands-Leuvens Screening Trial
NLST: National Lung Screening Trial
QALY: Quality-adjusted life year
USPSTF: US Preventive Services Task Force
VDT: Volume doubling time
