# Supplementary material for: Trade-off between benefits, harms and economic efficiency of low-dose CT lung cancer screening: a microsimulation analysis of nodule management strategies in a population-based setting
Source: BMC Med. 2017 Aug 25;15:162. doi: 10.1186/s12916-017-0924-3 (PMC5571665; doi:10.1186/s12916-017-0924-3)
Supplement: Additional file 1: — Microsimulation model components, modelling methods, input parameters and additional summaries of results. (PDF 3708 kb) [file 12916_2017_924_MOESM1_ESM.pdf]

Additional file 1

The first section of the current document contains a detailed description of the developed microsimulation model. The second part gives main results of model calibration and simulation.

Content

Tables 2
Figures 2
1. Methods 3
1.1. Modules of the microsimulation model 3
1.1.1. Population module 3
1.1.1.1. Other-cause mortality 3
1.1.2. Natural History module 3
1.1.3. Clinical detection and Survival module 4
1.1.4. Modelling details of the Natural History, Clinical detection and Survival modules 4
1.1.4.1. Onset of the first malignant cell: 4
1.1.4.2. Tumour growth 7
1.1.4.3. Modelling regional and distant stages of the disease progression 8
1.1.5. Screening module 9
1.1.5.1. Eligibility assessment 9
1.1.5.2. Screen-detection 9
1.1.5.3. Nodule management algorithms 9
1.1.5.4. Diagnostic work-up 10
1.1.5.5. Lung cancer survival 10
1.1.6. Life history module 11
1.1.6.1. False-positive findings 11
1.1.1.1. Overdiagnosed cases 11
1.1.1.2. Interval lung cancer 11
1.1.1.3. Radiation-induced cancer 11
1.1.7. Screening scenarios 11
1.1.8. Screening module: Parameters overview 12
1.2. Model calibration 12
1.3. Health economics 13
1.4. Sensitivity analysis 14
2. Results 15
2.1. Calibration 15
2.2. Benefits and harms of lung cancer screening for the baseline scenarios 18
2.3. Main outcomes and Cost-effectiveness of the 76 baseline screening scenarios. 21
2.4. Cost-effectiveness of the efficient screening scenarios in the sensitivity analyses 23
References ..... **Fehler! Textmarke nicht definiert.**

Tables

Table S1: Parameters for the long-term survival probability and the Weibull distributions for time period from clinical diagnosis to lung cancer death by cell type and stage at diagnosis [7] 4
Table S2: Parameters for the cumulative hazard functions 5
Table S3: Age boundaries ("a and b") for parametrization of age-dependent risk of the onset of the first malignant given in years by gender and histological class. 6
Table S4: Parameters for malignant conversion rate of initiated cells (μ)* by gender, period and cell type. 6
Table S5: Distribution of alpha parameters, α , for the growth rate applied in the Gompertz tumour growth function [5] 8
Table S6: Threshold values for volumes in mm^3 used to construct the log-Normal distributions in modelling of the disease progression. The parameters were fitted using data on lung cancer stages by Eberle 2015 [11] 9
Table S7: Parameters of the screening component 12
Table S8: Cost per unit: screening and no screening 13
Table S9: Lifetime treatment costs for patients diagnosed with lung cancer by cancer stages calculated for 50-75-15-9-NELSON-VDT400-V500. 14
Table S10: Benefits and harms of lung cancer screening for the baseline scenarios 18
Table S11: Benefits and harms of lung cancer screening for the baseline scenarios (continued) 19
Table S12: Main outcomes and Cost-effectiveness of the 76 baseline screening scenarios 21
Table S13: Cost-effectiveness of the efficient screening scenarios in the sensitivity analyses 23
Table S14: Comparison of the microsimulation model outcomes with the data from the NLST trial. 24

Figures

Figure S1: Diagnosed lung cancer cases, Men, 2010. 15
Figure S2: Diagnosed lung cancer cases, Men, 2011. 15
Figure S3: Diagnosed lung cancer cases, Men, 2012. 16
Figure S4: Diagnosed lung cancer cases, Women, 2010. 16
Figure S5: Diagnosed lung cancer cases, Women, 2011. 17
Figure S6: Diagnosed lung cancer cases, Women, 2012. 17
Figure S7: Accumulated lung cancer death cases 50-75-15-9-NELSON-VDT400-V500 vs. 50-75-15-9-NLST-GR10-D10 20
Figure S8: Accumulated lung cancer death cases 55-74-30-15-NELSON-VDT400-V500 vs. 55-74-30-15-NLST-GR10-D10 20

1. Methods

1.1. Modules of the microsimulation model

The model is of modular design and comprises of the following structural modules: Population, Natural History, Clinical Detection and Survival, Screening and Life History.

1.1.1. Population module

Population module creates a screening population with the given demographic structure and smoking patterns. The individuals in the simulated population were characterized by gender, age at model entry point and then defined by the age at the point of initial smoking, age at smoking cessation and the average number of cigarettes consumed per day. Smoking history determines the exposure to cigarette smoke (first hand), which along with age and gender governs age of death from other causes.

Smoking behaviour data were obtained from two national health surveys conducted between 2008 and 2012: the German Health Interview and Examination Survey for Adults (DEGS) and the German Health Update (GEDA)[1]. Due to the data availability, the demographic structure was taken from the year of 2012 [2]. Based on the smoking behaviour data and demographic structure, the population for the simulation was obtained via bootstrapping 10% of the German population. Smoking behaviours of current smokers were extrapolated over the course of a lifetime and during the modelled years the current smokers could quit smoking. The smoking cessation age was calculated by using the smoking cessation probabilities, which were assigned according to estimates obtained based on the data from the national health surveys.

1.1.1.1. Other-cause mortality

In the Population module an individual age of death from other causes than lung cancer is simulated based on age at entry the model, gender and the smoking status: never-, current- or former smoker. Five-year survival probabilities across age, gender and the smoking status were constructed based on the estimates obtained by Woloshin et al [3] and extrapolated using the recent life tables for the German population[2]. Other-cause mortality was introduced into the model as a competing risk and computed by applying the probability estimates and two random numbers (for each individual) which defined a five-year age interval in which the person may die from other causes and then the exact age of death within this interval.

1.1.2. Natural History module

The Natural History module simulates the development of lung cancer during individual life course. The sequence of events starts with onset of the first malignant cell, evolves through the progressive stages of lung cancer and ends with the death from the cancer.

The onset of the first malignant cell is simulated by using the biological two-stage clonal expansion (TSCE) model described by Moolgavkar and Luebeck [4], where age, gender and personal exposure to cigarette smoke are translated into the piecewise constant parameters of the hazard functions. Onset lung cancer is modelled as a competing risk between four histological types: small cell-, large cell-, squamous cell- and adenocarcinomas. For

each histological type, we drew an individual age at onset of carcinogenesis from a respective survival function. The histologic type that develops first is defined as the active cancer. We assume that 20% of adenocarcinomas are of type adenocarcinoma in situ[5]. Additionally, if the onset of cancer takes place, we assume a single malignant nodule per person.

The progression of the cancer is characterised by its growth, nodal involvement and occurrence of distant metastases. Threshold values of tumour volumes at the stages of nodal involvement and distant metastases depend on the histologic cancer type and are randomly drawn from log-Normal distributions. We applied a Gompertz function to model tumour growth over time[5]. This function determines the individual age at every stage of disease progression given the respective threshold volumes are reached (see section Modelling details of the Natural History, Clinical detection and Survival modules).

1.1.3. Clinical detection and Survival module

Clinical detection and Survival module simulates symptomatic detection of lung cancer, which includes age and tumour volume at the time of diagnosis, and age of death from lung cancer. The distribution of the tumour volumes at time of diagnosis is given by the log-Normal distribution; age at the time of diagnosis is analogously calculated by using the tumour growth function. Persons with clinical detection undergo diagnostic procedures which include PET CT, EBUS bronchoscopy and head MRI [6]. The diagnosis is assigned according to the TNM Classification of Malignant Tumours (TNM) by the Union for International Cancer Control (UICC). Treatment is not explicitly modelled, however, its effects are implicitly included in lung cancer survival function. The survival depends on the histological class and stage at the time of diagnosis and follows the Weibull distribution[7] (see Table 1). It is assumed that death from lung cancer occurs after the time of clinical diagnosis.

Table S1: Parameters for the long-term survival probability and the Weibull distributions for time period from clinical diagnosis to lung cancer death by cell type and stage at diagnosis [7].

**Histological class**	**Stage at Diagnosis**	**Long-term survival probability**	**Weibull distributions**	
			Mean	Shape
Squamous cell- carcinoma	I , II	0.180	2.419	0.573
Squamous cell-carcinoma	III , IV	0.060	0.752	0.641
Adeno- and Large cell-carcinoma	I , II	0.290	4.783	0.676
Adeno- and Large cell-carcinoma	III , IV	0.050	0.674	0.607
Small cell-carcinoma	I , II	0.080	1.049	0.727
Small cell-carcinoma	III , IV	0.010	0.507	0.738

1.1.4. Modelling details of the Natural History, Clinical detection and Survival modules

1.1.4.1. Onset of the first malignant cell:

Onset of the first malignant cell of each histological class is expressed by the biological two-stage clonal expansion (TSCE) model. The hazard rates and the survival probabilities are given by the equations below which were adopted from an R package "MIcrosimulation Lung Cancer (MILC) model" by Chrysanthopoulou AS. [8]

Hazard function for the development of the first malignant cell is described by [8]:

$$h(t) = \frac{\upsilon \mu X \left(e^{(\gamma + 2B)t} - 1\right)}{\gamma + B \left(e^{(\gamma + 2B)t} + 1\right)}$$

where X is total number of normal cells, υ is the normal cell initiation rate, μ is the malignant transformation rate, γ and B are piecewise constant parameters which are determined by:

$$\gamma = \alpha - \beta - \mu \text{ and } B = \frac{1}{2}\left(-\gamma + \sqrt{\gamma^2 + 4\alpha\mu}\right)$$

where α the cell division rate and β is the rate of programmed cell death.

For the hazard function, a cumulative hazard function than is constructed and given by [8]:

$$H(t) = \frac{\nu\mu X}{\gamma + B} * \left(-t + \frac{1}{B} * \log\left(\gamma + B + B * e^{(\gamma + 2B)t}\right)\right)$$

with

$$\alpha = \alpha_0 \left(1 + \alpha_1 q(t)^{\alpha_2}\right) \text{ and } \gamma = \gamma_0 \left(1 + \alpha_1 q(t)^{\alpha_2}\right),$$

where $q(t)$ is the average number of cigarettes consumed per day at age t and α_0 and γ_0 represent coefficients for never smokers. The parameters are given in Table 2.

Table S2: Parameters for the cumulative hazard functions

Parameter	Males	Females	Reference
Total number of normal cells (X)	10^7	10^7	[9]see Table 2 for CPS-II cohort.
Division rate of initiated cells<br>non-smokers(α_0)<br>smokers: α_1 ; α_2	 7.7<br>0.6 ; 0.22	 15.82<br>0.5 ; 0.32	
Piecewise constant parameters non-smokers (γ_0)	0.09	0.071	
The normal cell initiation rate non-smokers (ν_0)	= μ	= μ	
The normal cell initiation rate smokers (ν_1)	0	0.02	

For each histological class, the cumulative hazard functions are transformed into the survival functions which describe the time of the onset of lung cancer and are given by [8]:

$$S(t) = \exp\{-H(t)\} = \exp\left\{-\int_0^t h(x)\, dx\right\}$$

For each individual with onset carcinogenesis, the ages of onset of the first malignant cell of each histological type are drawn from the respective survival functions. The type of the active cancer and age of onset of carcinogenesis are modelled through competing risks between the four histological types and are determined by the histological type of the earliest cancer.

The life course is segmented into periods which are defined by age, gender and smoking status. Table 3 describes the division. The periods are bounded by age given by a and b, $0 < a < b < t_d$, where t_d depicts the age of death. Over these periods the survival functions are differently parameterized to express differences in the risk of onset of carcinogenesis. The parameters for the survival functions are given in Table 4 and are constant over the given period.

Table S3: Age boundaries ("a and b") for parametrization of age-dependent risk of the onset of the first malignant given in years by gender and histological class.

	Small cell-carcinoma	Large cell-carcinoma	Squamous cell-carcinoma	Adeno/AIS*-carcinoma
Male				
Age1 (a)	50.88	50.31	49.75	50.41
Age2 (b)	64.54	66.09	62.62	66.37
Female				
Age1 (a)	56.88	56.61	57.05	56.07
Age2 (b)	79.46	79.28	79.89	79.19

* adenocarcinoma *in situ*

Table S4: Parameters for malignant conversion rate of initiated cells (μ)* by gender, period and cell type.

	Small cell-carcinoma	Large cell-carcinoma	Squamous cell-carcinoma	Adeno/AIS-carcinoma
Male				
0 - a	2.13E-08	1.12E-08	2.70E-08	5.64E-08
a - b	2.67E-08	1.05E-08	4.14E-08	8.58E-08
b - 100	5.84E-08	2.07E-08	9.90E-08	1.26E-07
Female				
0 - a	4.51E-08	2.00E-08	3.96E-08	1.27E-07
a - b	7.37E-08	2.08E-08	7.46E-08	1.70E-07
b - 100	5.26E-08	1.71E-08	5.91E-08	4.60E-08

* The parameters were fitted using data on lung cancer incidence by Eberle 2015 and the German cancer registry [10,11].

Depending on the smoking status an individual life course can be divided into periods as follows. The periods are denoted by T_1, T_2, T_3 and T_4.

Never smoker:

For never smokers a life course is divided into three periods in which the survival function is parametrized with different malignant conversion rates (Table 4).

$$S(t) = \exp\left\{ - \int_0^{T_1} h(x)\,dx - \int_{T_1}^{T_2} h(x)\,dx - \int_{T_2}^{t_d} h(x)\,dx \right\},$$

with $T_1 = a$ and $T_1 = b$

Current smoker:

For current smokers a life course is divided into four periods which are defined by the age boundaries (as for never smokers) and age at start smoking. The age at smoking initiation can fall into any of the three periods and alter the parameterization for the hazard and survival functions over the periods following the time at smoking initiation as follows:

$$S(t) = \exp\left\{ - \int_0^{T_1} h(x)\,dx - \int_{T_1}^{T_2} h(x)\,dx - \int_{T_2}^{T_3} h(x)\,dx - \int_{T_3}^{t_d} h(x)\,dx \right\},$$

with T_{st} is age at start smoking, $0 < a < b < t; 0 < T_{st} < t_d$, and:

$$T_1 = \begin{cases} T_{st}, & T_{st} \le a \\ a, & T_{st} > a \end{cases}$$

$$T_2 = \begin{cases} a, & T_{st} \leq a \\ T_{st}, & a < T_{st} < b \\ b, & T_{st} \geq b \end{cases}$$

$$T_3 = \begin{cases} T_{st}, & T_{st} \geq b \\ b, & T_{st} < b \end{cases}$$

Former smoker:

For former smokers a life course is divided into five periods given by the age boundaries (as for non-smokers), age at smoking initiation and age at smoking cessation. The hazard and survival functions are respectively parameterized over the pre-smoking, smoking and post-smoking periods.

The survival functions for former smokers are described as follows:

$$S(t) = \exp\left\{- \int_0^{T_1} h(x)\,dx - \int_{T_1}^{T_2} h(x)\,dx - \int_{T_2}^{T_3} h(x)\,dx - \int_{T_3}^{T_4} h(x)\,dx - \int_{T_4}^{t_d} h(x)\,dx\right\},$$

with T_{st} age at initial cigarette smoking and T_q age of cessation, $0 < a < b < t_d; 0 < T_{st} < T_q < t_d$,

and:

$$T_1 = \begin{cases} T_{st}, & T_{st} \leq a \\ a, & T_{st} > a \end{cases}$$

$$T_2 = \begin{cases} T_q, & T_q < a \\ a, & T_{st} < a \text{ and } T_q \geq a \\ T_{st}, & a < T_{st} < b \end{cases}$$

$$T_3 = \begin{cases} a, & T_q \geq a \\ b, & T_{st} < b \text{ and } T_q \geq b \\ T_q, & T_{st} < b \text{ and } T_q < b \\ T_{st}, & T_{st} \geq b \end{cases}$$

$$T_4 = \begin{cases} b, & T_q < a \\ T_q, & T_{st} \geq b \end{cases}$$

1.1.4.2. Tumour growth

The following Gompertz function for tumour growth is applied:

$$V(t) = V_0 \cdot e^{\frac{\beta}{\alpha}\cdot(1-e^{-\alpha \cdot t})}$$

Where V_0 and $V(t)$ represent initial tumour volume and $V(t)$ tumour volume at time t, α and β are the location and scale parameters of the Gompertz distribution.

Maximum tumour volume V_{max} in the Gompertz function is given by:

$$V_{max} = V_0 \cdot e^{\frac{\beta}{\alpha}}$$

With a given V_{max} , the volume of the tumour developed over time t is expressed by:

$$V(t) = V_{max} \cdot \left(\frac{V_0}{V_{max}}\right)^{e^{(-\alpha \cdot t)}}$$

and time needed to reach volume $V(t)$ can be computed as:

$$t = \frac{\ln\left(\log_{\frac{V_0}{V_{max}}} \frac{V(t)}{V_{max}}\right)}{-\alpha},$$

where α is the growth rate which is drawn from lognormal distributions parameterized according to the histological class (see Table 5) [5].

Relationship between V_{max} and a set diameter is described by:

$$V_{max} = \frac{\pi}{6}(D)^3$$

where D is a given diameter.

Limits of diameters for V_{max} are fixed to 277 mm for all histological types except adenocarcinoma *in situ* for which the limit of diameter for V_{max} is set to 30 mm.

Table S 5: Distribution of alpha parameters, α , for the growth rate applied in the Gompertz tumour growth function [5].

Histological class	Distribution of alpha parameter	Mean (SD) Diameter at 0.5cm	Mean (SD) Diameter at 1.0cm	Mean (SD) Diameter at 1.5cm
Adeno/AIS-carcinoma	logN(-7.765, 0.5504)	187(160)	227(194)	260(222)
Large cell-carcinoma	logN(-6.59942, 0.68862)	61(61)	74(74)	85(85)
Small cell-carcinoma	logN(-5.44357, 0.611485)	19(16)	23(20)	26(23)
Squamous cell-carcinoma	logN(-6.6111, 0.7935)	65(72)	79(87)	90(100)

1.1.4.3. Modelling regional and distant stages of the disease progression

The disease progression is featured via tumour growth, nodal involvement (regional stage) and metastases (distant stage). It has been previously shown that with a Gompertzian tumour growth function, the disease progression through advanced stages over time are characterized by specific tumour volumes, location and presence of metastases can be well described by applying log-Normal distributions [8].

Threshold tumour volumes for regional and distant stages are drawn from log-Normal distributions constructed for each histological class i ($i = 1,2,3,4$) and stage j (j=regional, distant, clinical diagnosis) as $lognormal(\mu_{i,j}, \sigma^2_{i,j})$. If a person's threshold volume exceeds computed for her V_{max}, the corresponding cancer stage will not be reached during the lifetime of this person.

The threshold volumes across the histological classes and progression stages are given in the Table 6 below. The log-Normal distributions are constructed by transforming these volumes to mean and standard deviations of the $lognormal(\mu_{i,j}, \sigma^2_{i,j})$ distributions.

Table S6: Threshold values for volumes in mm^3 used to construct the log-Normal distributions in modelling of the disease progression. The parameters were fitted using data on lung cancer stages by Eberle 2015 [11]

Histological class	**Regional stage** Mean (SD)	**Distant stage** Mean (SD)	**Diagnosis before the regional stage** Mean (SD)	**Diagnosis after the regional stage** Mean (SD)
Small cell-carcinoma	610* (650)	4,710* (4,140)	4,787 (4,787)	9,031 (9,031)
Large cell-carcinoma	2,299 (2,299)	18,482 (18,482)	8,262 (8,262)	25,144 (25,144)
Squamous cell-carcinoma	8,466 (8,466)	74,610 (74,610)	24,458 (24,458)	56,418 (56,418)
Adeno/AIS-carcinoma	3,038 (3,038)	17,376 (17,376)	9,899 (9,899)	27,304 (27,304)

*adopted from McMahon et al 2012 [5]

1.1.5. Screening module

Screening module contains several structural components: eligibility assessment, screen-detection, nodule management (includes follow-up), diagnostic work-up and lung cancer survival.

1.1.5.1. Eligibility assessment

The eligibility criteria include qualifying age range, accumulated pack-years and number of years since cigarette cessation. Once eligible an individual undergoes a screen chest exam with LDCT.

1.1.5.2. Screen-detection

The probability of a screen-detection of a nodule depends on the presence of lung cancer and the sensitivity of the LDCT-test. The sensitivity of CT varies with nodule size and its location (Table 7). The location is considered of two types, central and peripheral, and varies with histological classes[5]. In the case of screen-detection of a nodule, the person proceeds through the nodule management algorithm. In the case of no detection, the person is scheduled for the next screening round.

1.1.5.3. Nodule management algorithms

The nodule management includes the nodule size assessment, classification of the screening test results and follow-up scans. The output of the nodule management predetermines whether the person goes through the work-up component or is scheduled for the next screening round. During simulation only one of the NLST and NELSON nodule management is switched depending on the screening scenario under evaluation.

See Figures 2 and 3 in the main text.

In the NELSON-line nodule management protocol, based on the assessed volume (V), the screening-detected nodule is classified as a negative ($V<V_{fup}$), positive ($V \geq V_{cut}$) or indeterminate result ($V_{fup} \leq V < V_{cut}$). Individuals with the negative initial results continue with annual screening. Individuals with the positive initial results undergo immediate diagnostic work-up. Persons with the indeterminate results undergo a follow-up imaging exam at three months after the initial screening. Results of the follow-up exam are determined by the nodule volume and the growth rate. The growth rate is defined by assessment of volume change (%) and volume

doubling time (VDT) [121]. At the follow-up the initial results are reclassified as positive if the nodule volume reaches or exceeds the cut-off volume (V_{cut}) and/or with VDT less than the threshold value (VDT_{cut}) defined by the scenario. The person with these results undergoes the work-up diagnostic procedures. If VDT is more than the threshold value, the person proceeds with the annual periodicity follow-up till the requirements for the positive result are met. Volumes at the follow-ups are compared with the volume of the initial screen-finding.

The NLST-like nodule management algorithm includes diametric assessment of the nodule size and a sequence of follow-up procedures where tumour growth is estimated as a change (%) in the nodule diameter relative to the result at the initial screening. Based on the assessed diameter (D) the nodule is placed into one of the three categories: negative ($D<D_{fup}$), positive intermediate ($D_{fup} \leq D < D_{cut}$) and positive ($D \geq D_{cut}$)[13]. People with negative initial results proceed to the next screening round. People with the positive initial results undergo diagnostic evaluation. Individuals with the intermediate initial results undergo a course of follow-up chest imaging exams with LDCT. The follow-up can occur with the fixed periodicity: at three, six and twelve months after the initial screening. The number of follow-up scans is managed according to the diameter of the nodule and its growth during the time between the initial screening and the follow-up exam. The growth is defined as a percentage increase in diameter and determined in screening scenario ($Growth_{cut}$). Measurement of growth is based on the comparison between the actual diameter and the diameter of the nodule found at the initial screen. In the follow-up course the diameter of 7 mm is the threshold diameter to undergo diagnostic evaluation. If at the first follow-up (at 3 months after the initial screening) no growth is detected, the person continues with an annual periodicity follow-up till the requirements for the positive result are met (D ≥7 mm) [13]. If the growth is present, the diameter is assessed. In case the diameter does not exceed the threshold, the person undergoes the next follow-up round within 6 month after the initial screening with assessment of the diameter. If the diameter of the nodule at the second follow-up (6 months) is over 7 mm, the person proceeds with the diagnostic work-up. In case the nodule size does not reach the threshold the person continues with the annual periodicity follow-up till the requirements for the positive result are met (D ≥7 mm). The cancer-indicating values for nodule size (V_{cut}, D_{cut}) and tumour growth ($VDT_{cut}, Growth_{cut}$) were taken from the trials and varied in the screening scenarios.

1.1.5.4. Diagnostic work-up

The diagnostic work-up component models a one-month long period when a patient undergoes a CT-supported biopsy to determine malignancy of the nodule and a head MRI (magnetic resonance imaging) and proceed with diagnosis. Screen-detected nodules are staged according to the TNM system based on the tumour diameter/volume and the progression state at time of diagnosis. During the diagnostic work-up a complication (pneumothorax) may occur, which is modelled as an age-dependent probability (see Table 7).

1.1.5.5. Lung cancer survival

Description is given in the main text.

---

[1] $VDT = \frac{\ln(2)\Delta t}{\ln(V_2)-\ln(V_1)}$,

where Δt is time in days between the initial screening and the follow-up exams, V_1 is the nodule volume at the time of initial screening, and V_2 is the volume at the follow-up

1.1.6. Life history module

For the screening and no screening scenarios, the *Life History* module calculates the final life scenario for each individual, providing the chronological sequence of events and final age of death along with the cause of death. The module also calculates events of false-positive cases, overdiagnosed cases, interval cancers and radiation induced cancer and deletes obsolete cases.

1.1.6.1. False-positive findings

False-positive findings of different sizes are simulated for people without lung cancer based on the outcomes of the clinical trials. For the NLST-based on nodule management algorithm, the number of follow-up scans and work-up of false-positive findings are estimated using the ratio of true positive to all positive findings obtained from the NLST trial results. For the NELSON nodule management follow-ups and work-ups of false-positive findings are estimated relative to the number of CT scans. The respective rates are calculated based on the results of the NELSON trial. Diagnostic work-up of false-positive finding includes a CT-supported biopsy, which may induce pneumothorax as a complication with the age-dependent probability (see Table 7). The false-positive findings are retroactively included into the model.

1.1.1.1. Overdiagnosed cases

A case of overdiagnosis is defined as an individual whose lung cancer is expected to be clinically diagnosed after her age of death from other causes but whose cancer is screen-detected before this age (de Koning, Harry J. et al. 2014).

1.1.1.2. Interval lung cancer

Interval lung cancer is defined as a cancer which is not initially screen-detected but is diagnosed in the time between scheduled screening exams [14]. The module incorporates two sources of interval lung cancer occurrence. The first is false-negative screening results, which can occur due to the nodule size-dependent sensitivity of CT scan. The second is the truly interval lung cancer, which develops and is diagnosed within the time interval between two screenings.

1.1.1.3. Radiation-induced cancer

Radiation-induced cancer death may occur in a 10–20-year period after the screening program. The risk is calculated as one radiation-induced cancer death per 2500 screened individuals who received 8 mSv in a 3-year period; these estimates are obtained based on the NLST trial [15].

1.1.7. Screening scenarios

Based on Table 1 in the main paper, name of a scenario contained specified population, nodule management protocol, thresholds for nodule size and nodule growth. The scenarios were additionally numbered from 1 to 76.

The scenarios that simulated NELSON-like and NLST-like nodule management protocols were 50-75-15-9-NELSON-VDT400-V500 and 55-74-30-15-NLST-GR10-D10.

1.1.8. Screening module: Parameters overview

Table S7: Parameters of the screening component

Parameter	NLST	NELSON	Reference
Sensitivity of screening CT exam for peripheral lesions.<br>Sensitivities for a central lesion of the same diameter are 25% lower (Probability of detection)	0.63 for $D \geq 1mm$<br>0.77 for $D \geq 4mm$<br>1.00 for $D \geq 8mm$		[16]
Specificity of screening CT exam	0.98		
Threshold nodule size for follow-up	$4mm \leq D < 10mm$	$50\ mm^3 \leq V < 500\ mm^3$	
Rate of "Stage II" at diagnosis: parameter for a binomial function which randomly defines whether the person at regional stage* is diagnosed with "Stage II"<br>at screening:<br>at no screening:	0.298701299<br>0.188034188		[13]
Complication rate at work up:<br>malignant nodule: $D \leq 2cm$<br>malignant nodule: $2 < D \leq 4cm$<br>malignant nodule: $D > 4cm$<br>benign nodule	 0.33<br>0.3<br>0.15<br>0.23		[17]
Long-term survival probability for stages I and II in the case the patients would die from lung cancer in the no screening scenario	0.4		[7]

*people at regional stage of cancer progression can be diagnosed either with stage II or stage III of TNM system.

1.2. Model calibration

The calibration process was performed in two steps. Firstly, for each lung cancer type mean and standard deviation of the log-Normal distributed threshold volumes of lymph nodes involvement (regional), distant metastases (distant) and clinical diagnosis were simultaneously calibrated to fit the German UICC data on diseases stage at time of diagnosis [11]. The parameters for the log-Normal distribution of the tumour volumes at time of clinical diagnosis differed depending on the disease stage progression: before and after the lymph nodes involvement (regional stage). Table 6 (section 1.1.4.3) presents the applied parameters in the columns "diagnosis before the regional stage" and "diagnosis after the regional stage". Data limitations allowed for the calibration of a limited number of parameters per cancer type. Therefore, we assumed that the mean and standard deviations of the threshold volumes are equal (see "Tumour growth" section).

Secondly, we simultaneously calibrated the age- and cancer type-dependent malignant conversion rates and age boundaries of the survival functions (derived from the hazard functions, see section 1.1.4.1). The outcomes of the microsimulation model (no screening scenario) were fitted to German age and cancer type specific annual incidental lung cancer cases of the period 2010-2012[11]. The second calibration step was done separately for males and females.

The Nelder-Mead Simplex method implemented in the R package "FME"[18] was used to minimize squared residuals in both calibration steps.

1.3. Health economics

The costs per unit were obtained using EBM (Unit assessment scale applied in the German healthcare) or DRG (Diagnosis Related Groups) codes and are summarized in Table 8. The model includes CT-guided needle biopsy-induced pneumothorax as a complication that leads to increased costs of the staging tests.

Table S8: Cost per unit: screening and no screening

	Procedure	Code EBM* or DRG**	Costs per unit	Reference
**Screening**				
**Screening**	Low dose CT Screening	No yet available	150€<br><br>Values for the sensitivity analyses:<br>I: 200€ ; II: 500€; III: 100€	Experts, [19]
**Diagnostic work-up and Staging**	CT-guided needle biopsy<br><br>Complication	EBM code 34505<br><br>DRG E76C	103€<br><br>2,976.88€	[20]<br><br>[21,17,22]
	Histology (pathology)	EBM code 19310	8.41€	[20]
	Head magnetic resonance imaging (MRI)	EBM code 34410	126.59€	[20]
	Medical contrast medium for MRI	EBM code 34452	46.55€	[20]
**No screening**				
**Diagnostic work-up and Staging**	Positron emission tomography (PET)	EBM code 34701	589.95€	[20]
	Endobronchial ultrasound-guided trans bronchial needle aspiration (EBUS-TBNA)	EBM code 13662 or 09315	988.00€	[20]
	Histology (pathology)	EBM code 19310	8.41€	[20]
	Head MRI	EBM code 34410	126.59€	[20]
	Medical contrast medium for MRT	EBM code 34452	46.55€	[20]

* Unit assessment scale applied in the German healthcare
** Diagnosis Related Groups

In the calculations of the total cost of screening we did not include lifetime lung cancer treatment costs and the costs for pharmaceuticals. The reason of omitting these expenditures is that there is partly available German data on life time costs stratified across ages and cancer stages and histology. Therefore we made the assumptions based on the literature. We based these assessments on data given by Mc Guire et al. [23] who calculated the costs of non-small cell lung cancer for Germany, France and England.

The average treatment costs for patients with metastatic disease were 27,932€ for the first year and 22,909€ for the second year after the diagnoses. We used these values to calculate costs for people with the advanced cancers in our model output. For that we calculated the mean survival of the patients with stage III and IV which is 1.100702 years (50-75-15-9-NELSON-VDT400-V500). Based on the mean survival and the average costs for each year (Mc. Guire) we calculated treatment costs of 26,698€ for advanced cancers (stage III and IV).

Mc Guire et al.[23] do not provide cost data for people with the early-staged cancers. In order to determine relevant costs for the early-staged cancers we took data on the lifetime costs for people with the early-staged cancers in the UK calculated by the British Department of Health [24]. Based on their estimates we calculated the ratio between the costs given for I-II and III-IV stages: (i) ratio of costs between III and I stages is used to define a base case scenario. Under these assumptions total treatment costs for Stage I and II are 30,101€ and for stage III

and IV 45,808€ (example for 50-75-15-9-NELSON-VDT400-V500). In order to obtain the costs for people with early-stage cancer in our model we applied these ratios to the costs calculated based on the mean survival and the average costs for late cancers [23]. The same calculations were performed for each of the six evaluated scenarios and scenarios of the sensitivity analysis.

1.4. Sensitivity analysis

Parameter uncertainty:

We varied the nodule size-dependent sensitivity parameters of LDCT exam within a range of ±20%. The long term survival probability for the screened individuals – who were diagnosed at screening with lung cancer in stage I or II and who would die of the cancer in the non-screening scenario – was tested for the range of values: 20%, 30%, 50% and 60%. We decreased adherence for the next years after the initial screening to 85%.

Additional scenarios:

We prolonged the period of the screening program and simulated ten years of annual screening for each of the evaluated scenarios. The cost per LDCT unit varied across three different scenarios (Table 8). Additionally, the total costs were analyzed for a hypothetical scenario (scenario 4) when staging tests at screening were the same as at clinical settings in no screening scenario.

Because treatment costs are based on different assumptions we tested possible impacts of the treatment costs in the sensitivity analyses. In the pessimistic scenario the costs for Stage I and II are based on the ratio of costs between stage IV and I (see Table 9, example is given for 50-75-15-9-NELSON-VDT400-V500). In the last years a few cost inducing pharmaceutical drugs for lung cancer treatment have been developed and introduced to the market [25]. It is possible that they were not taken into the calculations by Mc Guirre et al. To account for that we added the third scenario with lifetime costs for the patients with the advanced cancer of 77,702€ [26] (see Table 9).

Table S9: Lifetime treatment costs for patients diagnosed with lung cancer by cancer stages calculated for 50-75-15-9-NELSON-VDT400-V500.

Stages	Lifetime costs (British department of health[23])	Max Scenario (Cost Ratio IV / I)	Min Scenario (Cost Ratio III / I)	Scenario with new treatment options
Stage I	7,135.00 £	45,803.38 €**	31,960.12 €**	118,234.97 €**
Stage II	7,135.00 £	45,803.38 €**	31,960.12 €**	118,234.97 €**
Stage III	6,720.00 £	30,101.20 €*	30,101.20 €*	77,702.00 €
Stage IV	4,689.00 £	30,101.20 €*	30,101.20 €*	77,702.00 €

*calculated based on the mean survival and the average costs for late cancers [23]
** calculated based on the cost ratios multiplied with the costs for people with the advanced cancers

2. Results

2.1. Calibration

Figure S1: Diagnosed lung cancer cases, Men, 2010.

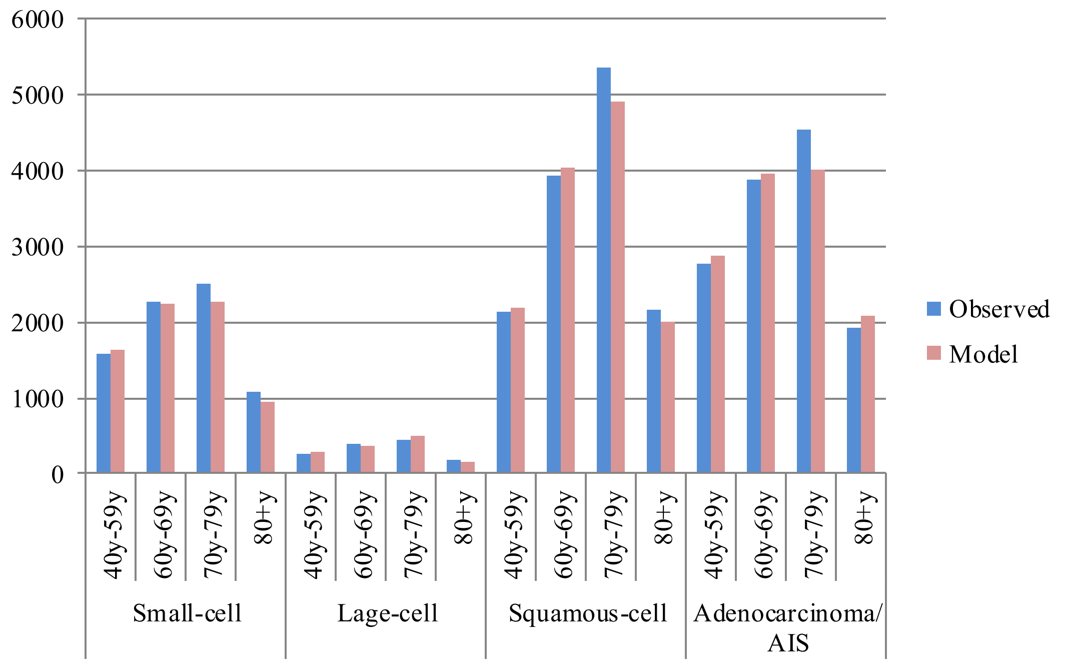

Figure S2: Diagnosed lung cancer cases, Men, 2011.

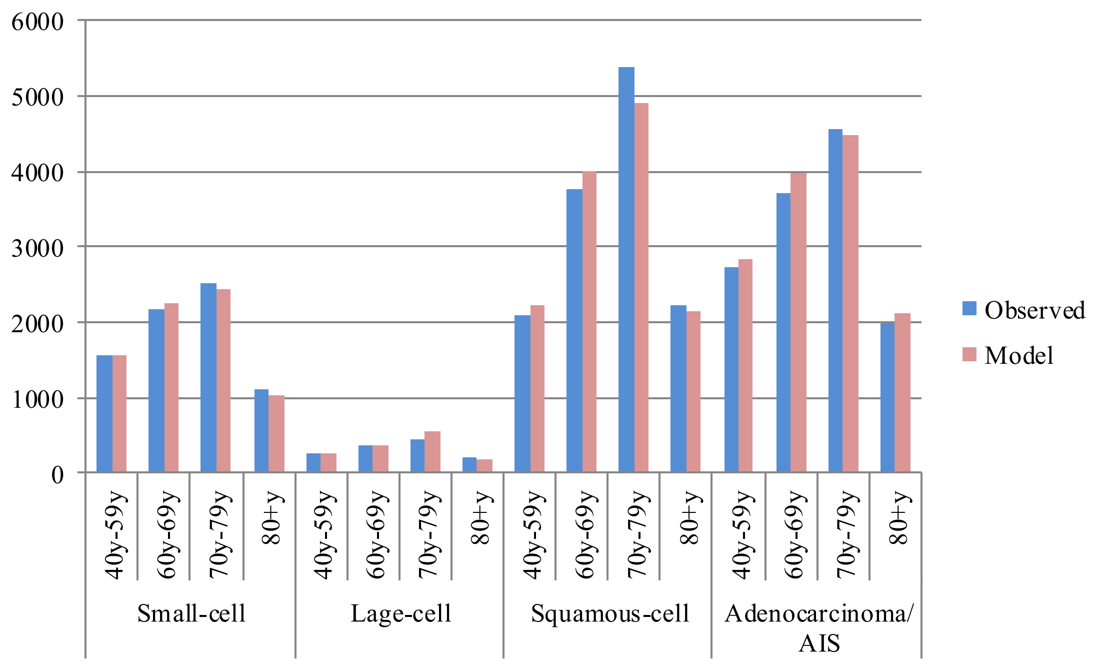

Figure S3: Diagnosed lung cancer cases, Men, 2012.

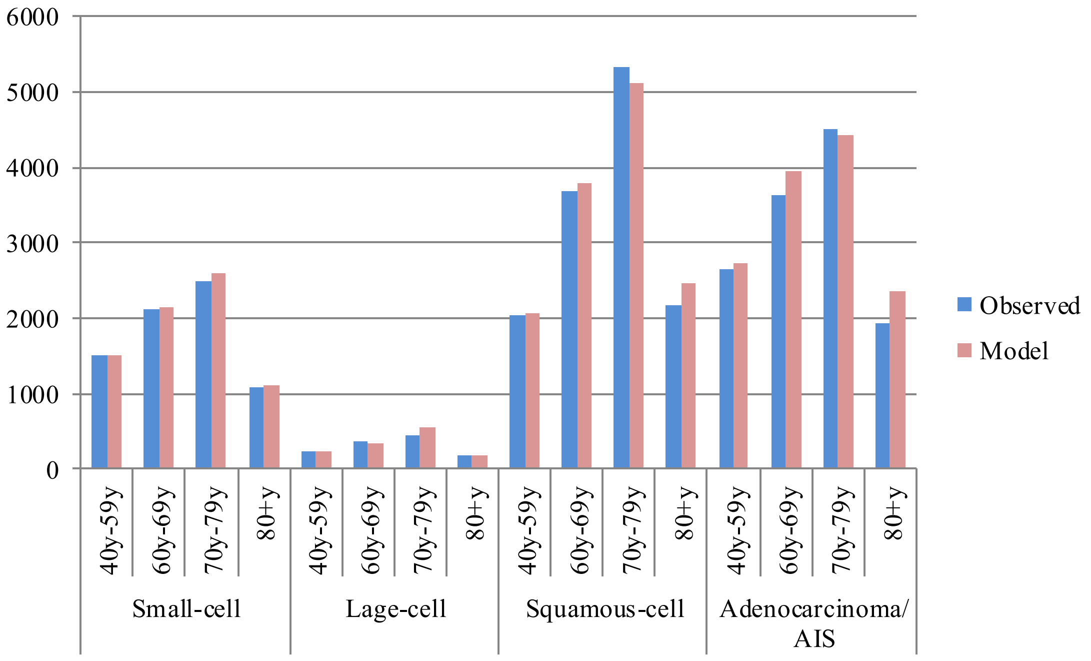

Figure S4: Diagnosed lung cancer cases, Women, 2010.

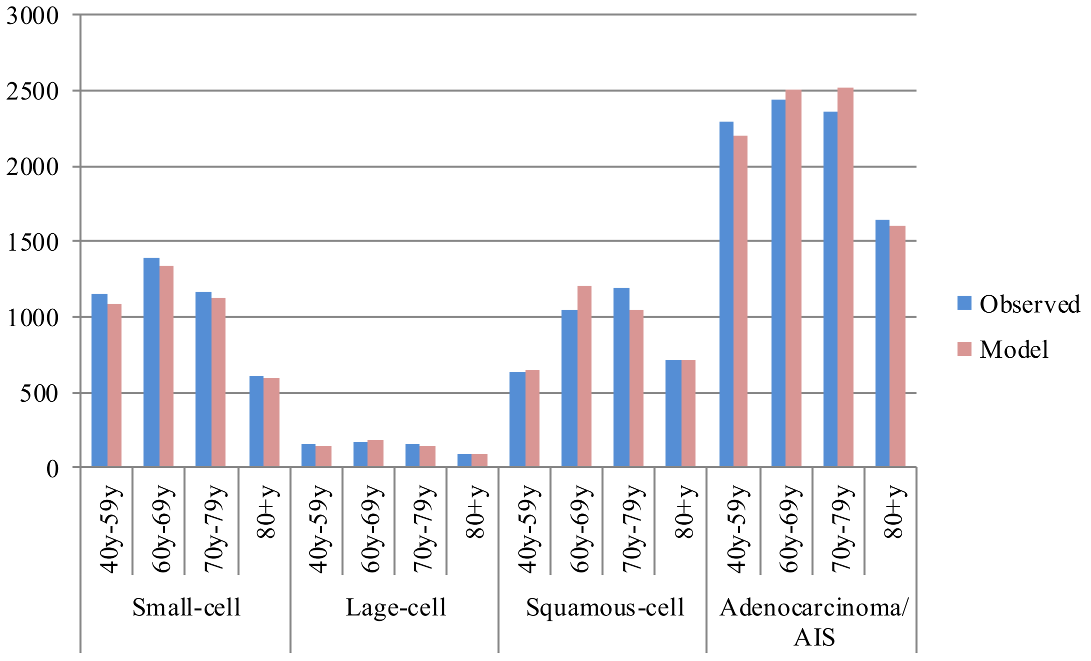

Figure S5: Diagnosed lung cancer cases, Women, 2011.

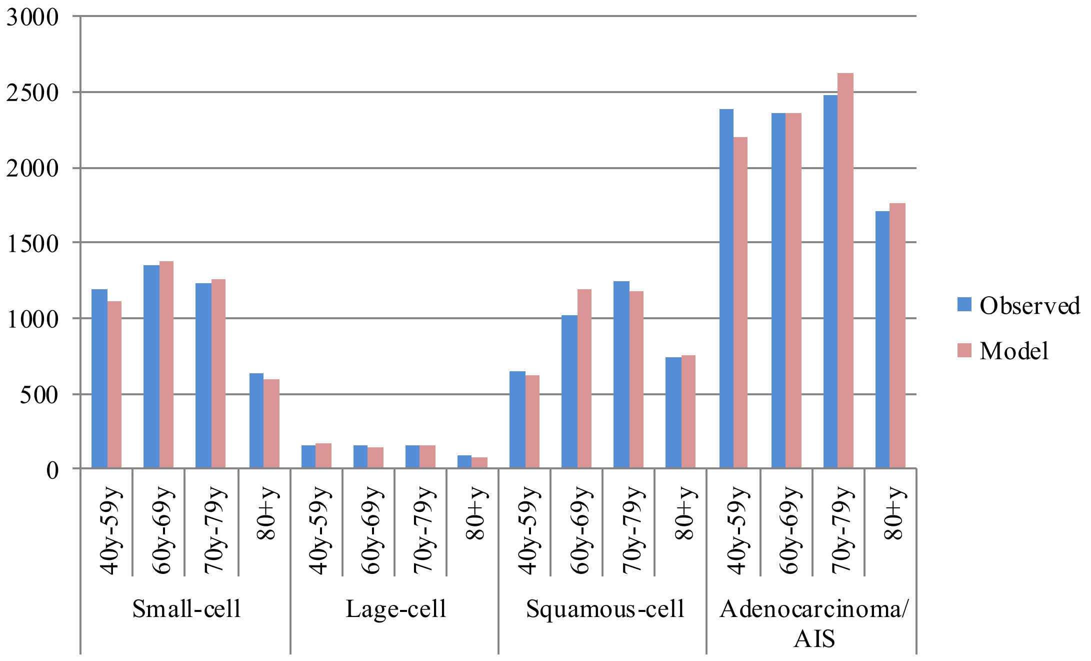

Figure S6: Diagnosed lung cancer cases, Women, 2012.

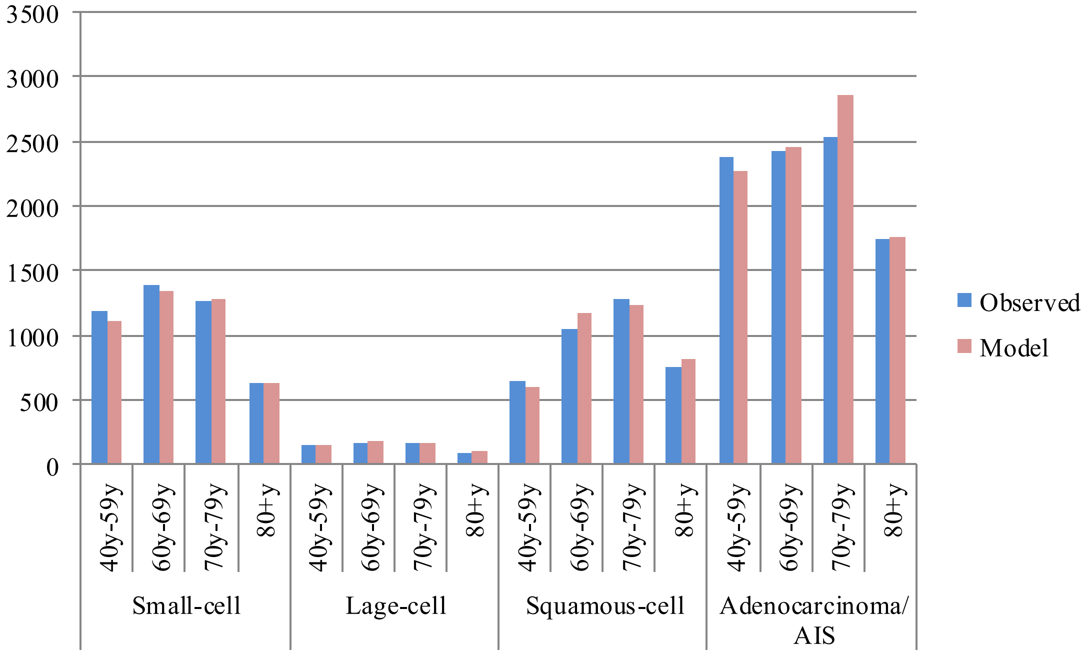

2.2. Benefits and harms of lung cancer screening for the baseline scenarios

Table S 10: Benefits and harms of lung cancer screening for the baseline scenarios

	50-75-15-9-NELSON-VDT400-V500	55-74-30-15-NELSON-VDT400-V500	50-75-15-9-NLST-GR10-D10	55-74-30-15-NLST-GR10-D10
Number of people screened	7,431,345	4,373,484	7,431,345	4,373,484
Screening outcomes				
Lung Cancer Findings	179,504	126,910	181,468	128,484
Screen detection Stage I	114,379	79,598	109,608	76,595
Screen detection Stage II	13,254	9,583	14,774	10,633
Screen detection Stage III	31,447	22,698	34,879	25,024
Screen detection Stage IV	20,424	15,031	22,207	16,232
Stage III, %	17.52	17.89	19.22	19.48
Stage IV, %	11.38	11.84	12.24	12.63
Total Cases Detected at an Early Stage	127,633	89,181	124,382	87,228
Total Cases Detected at an Early Stage, %	71.10	70.27	68.54	67.89
Small-cell carcinoma	10,048	6,601	10,528	6,915
Large-cell carcinoma	5,003	3,490	5,106	3,560
Squamous-cell carcinoma	42,688	29,549	43,182	29,873
Adenocarcinoma	92,003	64,423	91,697	64,209
Adenocarcinoma *in situ*	29,762	22,847	30,955	23,927
Small-cell carcinoma, %	5.60	5.20	5.80	5.38
Large-cell carcinoma, %	2.79	2.75	2.81	2.77
Squamous-cell carcinoma, %	23.78	23.28	23.80	23.25
Adenocarcinoma, %	51.25	50.76	50.53	49.97
Adenocarcinoma *in situ*, %	16.58	18.00	17.06	18.62
False-Positive Findings	262,311	185,356	4,531,519	3,208,432
False-Positive Findings of all screen detected findings	59.37	59.36	96.15	96.15
Interval cancer: False Negative Detection	33,111	21,763	32,101	21,106
Small-cell carcinoma	15,894	10,275	15,464	9,994
Large-cell carcinoma	1,576	1,044	1,520	1,005
Squamous-cell carcinoma	11,449	7,676	11,097	7,440
Adenocarcinoma	4,174	2,754	4,006	2,660
Adenocarcinoma *in situ*	18.00	14.00	14.00	7.00
Interval Cancer Stage I	5,363	3,556	5,147	3,413
Interval Cancer Stage II	1,810	1,211	1,765	1,182
Interval Cancer Stage III	8,745	5,619	8,467	5,439
Interval Cancer Stage IV	17,193	11,377	16,722	11,072
True Interval cancer	10,232	6,638	10,232	6,638
Small-cell carcinoma	8,494	5,504	8,494	5,504
Large-cell carcinoma	201	142	201	142
Squamous-cell carcinoma	1,517	981	1,517	981
Adenocarcinoma	20.00	11.00	20.00	11.00
Adenocarcinoma *in situ*	0.00	0.00	0.00	0.00
Interval Cancer Stage I	818	535.00	818	535
Interval Cancer Stage II	389	263	389	263
Interval Cancer Stage III	2,594	1,683	2,594	1,683
Interval Cancer Stage IV	6,431	4,157	6,431	4,157
Small-cell carcinoma, % of interval cancers	56.27	55.56	56.59	55.86
Stage IV, % of interval cancers	54.50	54.70	54.69	54.89
Clinical Detection	771,760	435,763	770,918	435,228
Clinical Detection: onset of cancer before the end of screening	208,902	137,329	208,060	136,794
All detected cancers: onset of cancer before the end of screening	388,406	264,239	389,528	265,278
Overdiagnosis	31,005	23,772	31,385	24,260
Overdiagnosis, % of screening detected cases	17.27	18.73	17.30	18.88
Small-cell carcinoma	51.00	33.00	52.00	35.00
Large-cell carcinoma	147	110.00	144	105
Squamous-cell carcinoma	1,683	1,201	1,606	1,155
Adenocarcinoma	6,429	4,795	5,934	4,461
Adenocarcinoma *in situ*	22,695	17,633	23,649	18,504
Adenocarcinoma *in situ*, %	73.20	74.18	75.35	76.27
Overdiagnosis Stage I	19,722	14,434	19,282	14,369
Overdiagnosis Stage II	2,368	1,919	2,603	2,090
Overdiagnosis Stage III	5,613	4,573	6,141	4,916
Overdiagnosis Stage IV	3,302	2,846	3,359	2,885
Radiation-induced Lung Cancer Deaths	2,390	1,329	2,388	1,328
No screening scenario				
Clinical Detection no screening	919,585	538,385	919,585	538,385
Clinical Detection Stage 1	132,312	77,379	132,312	77,379
Clinical Detection Stage 2	56,328	33,417	56,328	33,417
Clinical Detection Stage 3	235,578	138,090	235,578	138,090
Clinical Detection Stage 4	495,367	289,499	495,367	289,499
Clinical Detection: onset of cancer before the end of screening	356,727	239,951	356,727	239,951

Table S 11: Benefits and harms of lung cancer screening for the baseline scenarios (continued)

	50-75-15-9-NELSON-VDT400-V500	55-74-30-15-NELSON-VDT400-V500	50-75-15-9-NLST-GR10-D10	55-74-30-15-NLST-GR10-D10
Clinical detection during the first five years: Histological class	152,040	102,786	152,040	102,786
Small-cell carcinoma	35,472	23,234	35,472	23,234
Large-cell carcinoma	6,265	4,321	6,265	4,321
Squamous-cell carcinoma	48,009	32,720	48,009	32,720
Adenocarcinoma	59,673	40,630	59,673	40,630
Adenocarcinoma *in situ*	2,621	1,881	2,621	1,881
Clinical Detection Stage 1	22,129	15,039	22,129	15,039
Clinical Detection Stage 2	9,129	6,247	9,129	6,247
Clinical Detection Stage 3	38,797	26,004	38,797	26,004
Clinical Detection Stage 4	81,985	55,496	81,985	55,496
**Deaths from lung cancer**				
Death from lung cancer: screening	763,653	442,246	764,847	443,061
Death from lung cancer: onset of cancer before the end of screening	275,110	184,150	276,304	184,965
Death from lung cancer: no screening	800,040	467,246	800,040	467,246
Death from lung cancer: no screening (onset of cancer before the end of screening)	311,497	209,150	311,497	209,150
**Mortality reduction vs no screening, %**	**11.68**	**11.95**	**11.30**	**11.56**
**Benefits of screening vs no screening**				
**Averted death vs no screening**	**36,387**	**25,000**	**35,193**	**24,185**
Life years gained vs no screening	541,697	356,262	525,811	345,918
**Life years gained vs no screening: 3% discount**	**355,348**	**236,371**	**346,100**	**230,284**
Life years gained vs no screening: 1.5% discount	435,161	288,028	423,115	280,136
**Healthcare resources for the screening program**				
Number of Screen exams	29,969,925	16,660,175	29,955,605	16,650,031
Number of Follow-up scans	2,781,924	1,525,291	4,011,903	2,839,342
Number of Follow-up scans: malignant nodules	100,296	71,876	157,843	110,595
Number of Work-ups	441,815	312,266	945,678	669,564
Number of Work-ups: malignant nodules	171,637	121,317	173,250	122,633
Number of Complications	117,474	82,898	233,375	165,097
**Efficiency of screening**				
Detected cancer per 1000 scans	5.99	7.62	6.06	7.72
Interval cancers per 1000 screen-scans	1.45	1.70	1.41	1.67
Lung cancer deaths per 1000 screen-scans: onset of cancer before the end of screening	9.18	11.05	9.22	11.11
Averted lung cancer deaths vs no screening per 1000 screen-scans	1.21	1.50	1.17	1.45
Life years gained (3% discount) vs no screening per 1000 screen-scans	11.86	14.19	11.55	13.83
**Health economics outcomes of screening vs no screening**				
Total costs (discounted)	29363651302	17776335686	29885787583	18223237851
Total costs: no screening(discounted)	21900234274	13183590963	21900234274	13183590963
Additional costs vs. no screening (discounted)	7463417028	4592744723	7985553310	5039646887
ACER: Costs (including life time treatment costs) per Life Year Gained (uniform discounting) vs no screening	21,003	19,430	23,072	21,884
**Cost categories (Discounted 3%)**				
Screening scans	4,243,729,151	2,355,013,913	4,241,738,728	2,353,604,382
Work-up total malignant	211,365,526	149,013,209	212,913,711	150,313,048
Complication	178,066,109	125,439,507	179,259,484	126,453,389
Without complication	33,299,417	23,573,702	33,654,226	23,859,659
Follow-up malignant	15,044,522	10,781,399	23,676,532	16,589,331
False-Positive Work-up total	201,146,272	142,104,689	580,683,348	411,030,986
Complication	179,465,545	126,787,810	518,093,885	366,727,652
Without complication	21,680,726	15,316,878	62,589,462	44,303,334
False-Positive Follow-up	385,177,551	208,247,816	550,582,775	389,724,592
Interval cancer: False-Negative Detection	55,046,074	36,158,088	53,381,576	35,078,201
True Interval cancer	16,964,750	10,988,952	16,964,750	10,988,952
Treatment	24,235,177,456	14,864,027,620	24,205,846,163	14,855,908,359

Figure S7: Accumulated lung cancer death cases 50-75-15-9-NELSON-VDT400-V500 vs. 50-75-15-9-NLST-GR10-D10

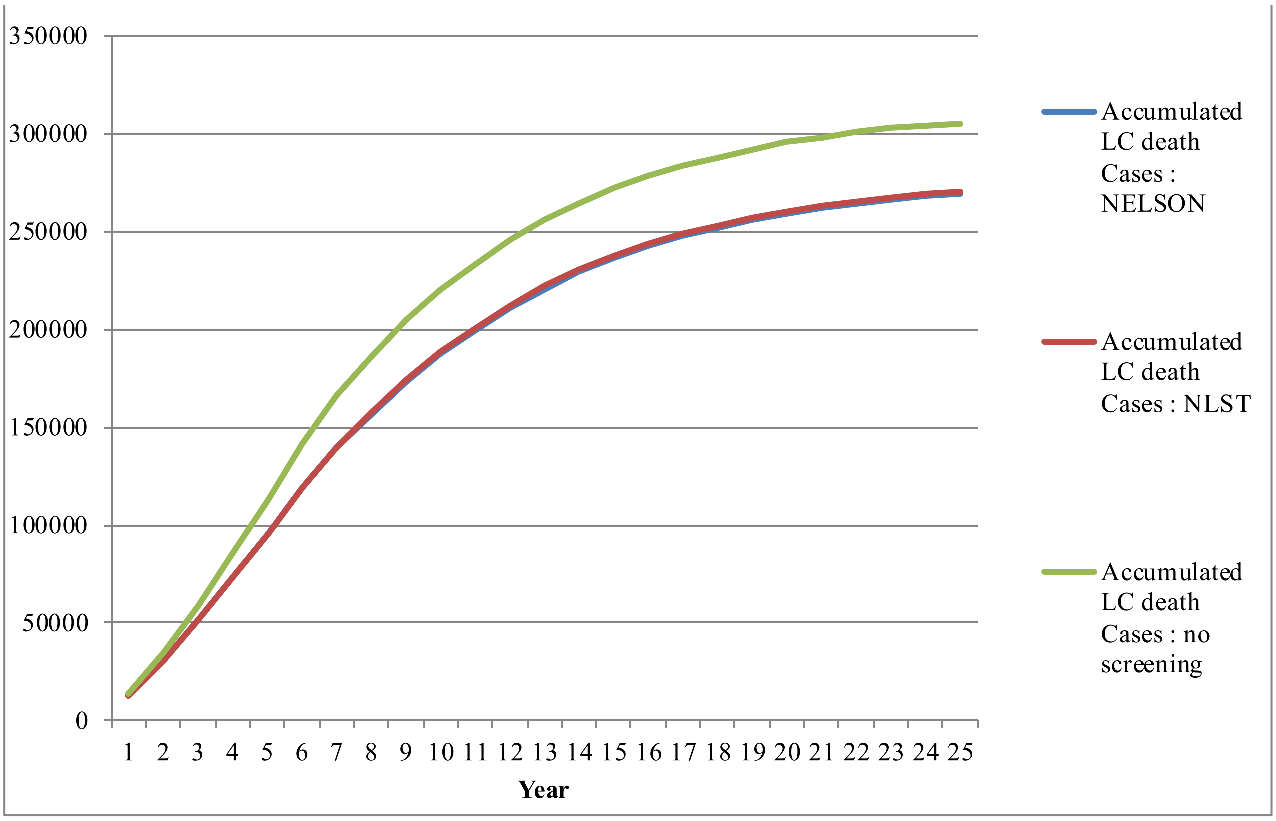

Figure S8: Accumulated lung cancer death cases 55-74-30-15-NELSON-VDT400-V500 vs. 55-74-30-15-NLST-GR10-D10

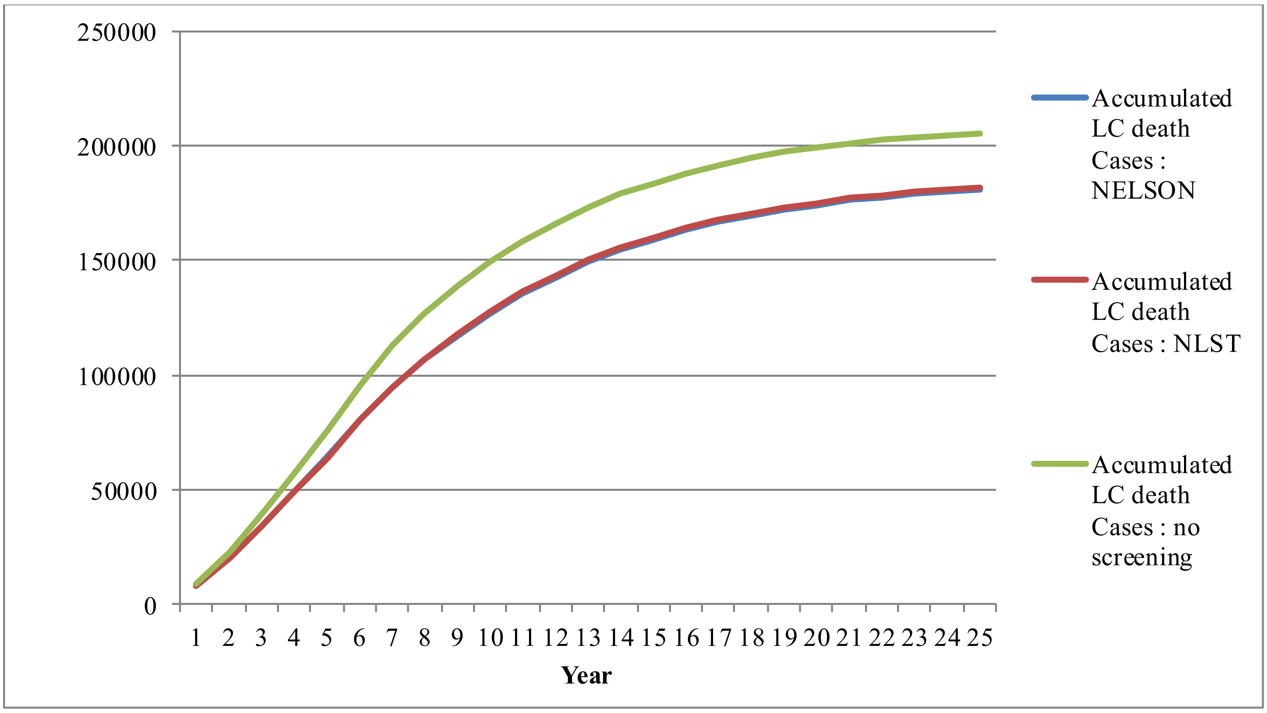

2.3. Main outcomes and Cost-effectiveness of the 76 baseline screening scenarios.

Table S12: Main outcomes and Cost-effectiveness of the 76 baseline screening scenarios.

Scenario	Scenario characteristics	Detected cancers at an early stage (I/II), %	Reduction in lung cancer mortality, %	Lung cancer deaths averted	Discounted life years gained	Interval cancer cases	Over diagnosed cases	Over diagnosis, %	Discounted total cost, million Euro	Discounted additional costs vs no screening, million Euro	Cost per life years gained vs no screening (uniform discounting) Euro	Discounted cost per lung cancer death averted vs no screening, Euro	ICER vs the previous efficient scenario, Euro per LYG	ICER vs the previous efficient scenario, Euro per averted lung cancer death
Scenario 65	55-75-40-10-NELSON-VDT300-none	67.31	9.95	14,373	133,222	23,057	6,733	9.48	10,892,118,387	2,231,946,546	16,754	155,287	16,754	155,287
Scenario 64	55-75-40-10-NELSON-VDT400-none	67.95	10.65	15,395	140,490	21,367	9,184	11.73	11,056,787,394	2,396,615,552	17,059	155,675	not efficient	161,124
Scenario 66	55-75-40-10-NELSON-VDT600-none	68.25	11.00	15,891	143,763	20,406	12,036	14.35	11,211,978,121	2,551,806,280	17,750	160,582	not efficient	not efficient
Scenario 75	55-75-40-10-NLST-GR12.5-none	67.50	11.37	16,430	147,652	19,629	15,341	17.20	11,716,673,226	3,056,501,385	20,701	186,032	not efficient	not efficient
Scenario 73	55-75-40-10-NLST-GR10-none	67.91	11.52	16,638	149,484	19,570	16,074	17.75	11,766,309,036	3,106,137,195	20,779	186,689	not efficient	not efficient
Scenario 74	55-75-40-10-NLST-GR7.5-none	68.20	11.63	16,798	150,829	19,514	16,812	18.34	11,811,094,697	3,150,922,856	20,891	187,577	not efficient	not efficient
Scenario 76	55-75-40-10-NLST-Dfup5	67.08	11.26	16,270	151,944	20,278	16,476	18.90	11,712,212,808	3,052,040,967	20,087	187,587	not efficient	not efficient
Scenario 67	55-75-40-10-NELSON-Vfup80	68.43	11.49	16,604	154,199	20,558	16,389	18.92	11,381,642,165	2,721,470,324	17,649	163,905	not efficient	not efficient
Scenario 72	55-75-40-10-NLST-GR12.5-D10	66.61	11.48	16,594	154,561	19,424	17,268	19.07	11,789,031,396	3,128,859,555	20,243	188,554	not efficient	not efficient
Scenario 70	55-75-40-10-NLST-GR10-D11	66.84	11.59	16,741	155,308	19,429	17,366	19.11	11,800,360,566	3,140,188,725	20,219	187,575	not efficient	not efficient
Scenario 68	55-75-40-10-NLST-GR10-D10	67.69	11.75	16,976	157,701	19,409	17,467	19.19	11,817,384,038	3,157,212,197	20,020	185,981	not efficient	not efficient
Scenario 59	55-75-40-10-NELSON-VDT400-V750	68.73	11.90	17,201	158,585	19,883	16,598	18.63	11,416,918,590	2,756,746,749	17,383	160,267	not efficient	not efficient
Scenario 71	55-75-40-10-NLST-GR7.5-D10	68.35	11.90	17,200	159,297	19,404	17,647	19.30	11,838,944,522	3,178,772,681	19,955	184,812	not efficient	not efficient
Scenario 69	55-75-40-10-NLST-GR10-D9	68.50	11.91	17,212	159,963	19,392	17,575	19.27	11,834,207,265	3,174,035,423	19,842	184,408	not efficient	not efficient
Scenario 62	55-75-40-10-NELSON-VDT300-V500	69.95	12.14	17,542	161,967	19,866	17,162	19.09	11,459,390,353	2,799,218,512	17,283	159,572	not efficient	not efficient
Scenario 58	55-75-40-10-NELSON-VDT400-V500	70.07	12.16	17,564	162,073	19,862	17,221	19.14	11,465,748,287	2,805,576,446	17,311	159,734	not efficient	not efficient
Scenario 63	55-75-40-10-NELSON-VDT600-V500	70.30	12.19	17,608	162,281	19,857	17,656	19.49	11,491,382,892	2,831,211,051	17,446	160,791	not efficient	not efficient
Scenario 61	55-75-40-10-NELSON-VDT400-V400	70.71	12.27	17,726	163,470	19,857	17,573	19.42	11,491,130,432	2,830,958,591	17,318	159,707	not efficient	not efficient
Scenario 60	55-75-40-10-NELSON-VDT400-V300	71.35	12.38	17,889	164,864	19,854	17,892	19.69	11,515,704,974	2,855,533,133	17,321	159,625	19,707	184,009
Scenario 8	55-74-30-15-NELSON-VDT300-none	67.41	9.72	20,335	192,747	33,009	9,106	9.12	16,964,229,032	3,780,638,069	19,615	185,918	not efficient	not efficient
Scenario 7	55-74-30-15-NELSON-VDT400-none	68.13	10.47	21,908	204,456	30,527	12,562	11.37	17,202,833,077	4,019,242,114	19,658	183,460	not efficient	not efficient
Scenario 27	55-80-30-15-NELSON-VDT300-none	67.52	10.10	23,029	207,468	38,212	11,724	10.14	17,709,501,637	4,159,369,822	20,048	180,614	not efficient	not efficient
Scenario 9	55-74-30-15-NELSON-VDT600-none	68.49	10.82	22,633	209,464	29,157	16,599	14.01	17,425,676,762	4,242,085,799	20,252	187,429	not efficient	not efficient
Scenario 18	55-74-30-15-NLST-GR12.5-none	67.86	11.21	23,437	215,599	28,049	21,270	16.88	18,086,156,020	4,902,565,056	22,739	209,181	not efficient	not efficient
Scenario 16	55-74-30-15-NLST-GR10-none	68.21	11.35	23,747	218,277	27,972	22,232	17.39	18,153,904,252	4,970,313,288	22,771	209,303	not efficient	not efficient
Scenario 17	55-74-30-15-NLST-GR7.5-none	68.46	11.46	23,962	220,191	27,900	23,237	17.96	18,215,134,331	5,031,543,367	22,851	209,980	not efficient	not efficient
Scenario 26	55-80-30-15-NELSON-VDT400-none	68.24	10.93	24,917	220,616	35,048	16,282	12.62	18,002,132,447	4,452,000,631	20,180	178,673	not efficient	not efficient
Scenario 19	55-74-30-15-NLST-Dfup5	67.28	11.07	23,152	221,728	28,955	22,938	18.63	18,078,388,416	4,894,797,453	22,076	211,420	not efficient	not efficient
Scenario 10	55-74-30-15-NELSON-Vfup80	68.62	11.29	23,620	224,880	29,359	22,740	18.59	17,662,309,284	4,478,718,321	19,916	189,616	not efficient	not efficient
Scenario 15	55-74-30-15-NLST-GR12.5-D10	66.85	11.31	23,646	225,756	27,773	24,000	18.76	18,184,707,034	5,001,116,071	22,153	211,499	not efficient	not efficient
Scenario 28	55-80-30-15-NELSON-VDT600-none	68.57	11.35	25,871	226,617	33,118	21,861	15.63	18,293,791,730	4,743,659,914	20,932	183,358	not efficient	not efficient
Scenario 13	55-74-30-15-NLST-GR10-D11	67.05	11.39	23,831	226,640	27,773	24,145	18.81	18,200,613,216	5,017,022,253	22,136	210,525	not efficient	not efficient
Scenario 11	55-74-30-15-NLST-GR10-D10	67.89	11.56	24,185	230,284	27,744	24,260	18.88	18,223,237,851	5,039,646,887	21,885	208,379	not efficient	not efficient

Scenario 2	55-74-30-15-NELSON-VDT400-V750	68.89	11.69	24,442	231,099	28,431	22,874	18.22	17,705,725,213	4,522,134,250	19,568	185,015	not efficient	not efficient
Scenario 14	55-74-30-15-NLST-GR7.5-D10	68.57	11.72	24,512	232,766	27,736	24,493	18.99	18,253,498,654	5,069,907,690	21,781	206,834	not efficient	not efficient
Scenario 37	55-80-30-15-NLST-GR12.5-none	67.77	11.72	26,719	232,797	31,764	28,055	18.78	19,112,331,877	5,562,200,062	23,893	208,174	not efficient	not efficient
Scenario 12	55-74-30-15-NLST-GR10-D9	68.72	11.73	24,527	233,603	27,721	24,418	18.97	18,247,633,560	5,064,042,597	21,678	206,468	not efficient	not efficient
Scenario 35	55-80-30-15-NLST-GR10-none	68.13	11.89	27,092	235,833	31,657	29,431	19.39	19,199,988,990	5,649,857,174	23,957	208,543	not efficient	not efficient
Scenario 5	55-74-30-15-NELSON-VDT300-V500	70.15	11.94	24,973	236,226	28,407	23,689	18.68	17,767,037,281	4,583,446,318	19,403	183,536	not efficient	not efficient
Scenario 1	55-74-30-15-NELSON-VDT400-V500	70.27	11.95	25,000	236,371	28,401	23,772	18.73	17,776,335,686	4,592,744,723	19,430	183,710	not efficient	not efficient
Scenario 6	55-74-30-15-NELSON-VDT600-V500	70.52	11.98	25,059	236,665	28,392	24,434	19.12	17,814,338,122	4,630,747,159	19,567	184,794	not efficient	not efficient
Scenario 36	55-80-30-15-NLST-GR7.5-none	68.34	12.00	27,350	238,024	31,556	30,922	20.08	19,284,015,000	5,733,883,184	24,090	209,648	not efficient	not efficient
Scenario 4	55-74-30-15-NELSON-VDT400-V400	70.90	12.06	25,223	238,424	28,393	24,278	19.03	17,812,380,227	4,628,789,264	19,414	183,515	not efficient	not efficient
Scenario 3	55-74-30-15-NELSON-VDT400-V300	71.58	12.18	25,467	240,626	28,389	24,767	19.32	17,849,042,023	4,665,451,059	19,389	183,196	not efficient	not efficient
Scenario 38	55-80-30-15-NLST- Dfup5	66.76	11.67	26,589	240,683	32,739	31,090	21.03	19,160,111,768	5,609,979,952	23,309	210,989	not efficient	not efficient
Scenario 29	55-80-30-15-NELSON-Vfup80	68.00	11.89	27,104	244,027	33,178	30,722	20.93	18,625,060,345	5,074,928,530	20,797	187,239	not efficient	not efficient
Scenario 34	55-80-30-15-NLST-GR12.5-D10	66.35	11.89	27,105	244,796	31,380	32,411	21.15	19,281,610,562	5,731,478,747	23,413	211,455	not efficient	not efficient
Scenario 32	55-80-30-15-NLST-GR10-D11	66.56	11.98	27,308	245,671	31,391	32,590	21.19	19,299,330,176	5,749,198,361	23,402	210,532	not efficient	not efficient
Scenario 30	55-80-30-15-NLST- GR10-D10	67.37	12.16	27,706	249,634	31,348	32,764	21.27	19,325,894,027	5,775,762,212	23,137	208,466	not efficient	not efficient
Scenario 21	55-80-30-15-NELSON-VDT400-V750	68.19	12.28	27,987	250,445	32,125	30,739	20.47	18,661,518,852	5,111,387,037	20,409	182,634	not efficient	not efficient
Scenario 33	55-80-30-15-NLST-GR7.5-D10	68.04	12.31	28,062	252,243	31,338	33,098	21.40	19,361,758,630	5,811,626,814	23,040	207,100	not efficient	not efficient
Scenario 31	55-80-30-15-NLST-GR10-D9	68.18	12.32	28,080	253,130	31,313	32,991	21.38	19,354,607,603	5,804,475,788	22,931	206,712	not efficient	not efficient
Scenario 24	55-80-30-15-NELSON-VDT300-V500	69.49	12.55	28,596	256,006	32,094	31,929	21.02	18,741,364,602	5,191,232,786	20,278	181,537	not efficient	not efficient
Scenario 20	55-80-30-15-NELSON-VDT400-V500	69.61	12.56	28,625	256,159	32,086	32,050	21.08	18,752,973,157	5,202,841,342	20,311	181,759	not efficient	not efficient
Scenario 25	55-80-30-15-NELSON-VDT600-V500	69.89	12.59	28,694	256,478	32,077	32,983	21.53	18,803,698,202	5,253,566,386	20,483	183,089	not efficient	not efficient
Scenario 23	55-80-30-15-NELSON-VDT400-V400	70.25	12.67	28,877	258,339	32,076	32,752	21.42	18,798,713,288	5,248,581,472	20,317	181,756	not efficient	not efficient
Scenario 22	55-80-30-15-NELSON-VDT400-V300	70.95	12.80	29,165	260,807	32,071	33,473	21.76	18,846,402,156	5,296,270,341	20,307	181,597	not efficient	216,454
Scenario 46	50-75-15-9-NELSON-VDT300-none	67.40	9.68	30,147	295,093	48,838	13,432	9.09	28,347,809,378	6,447,575,105	21,849	213,871	not efficient	not efficient
Scenario 45	50-75-15-9-NELSON-VDT400-none	68.06	10.27	31,994	308,862	45,891	17,943	11.18	28,650,791,510	6,750,557,237	21,856	210,994	not efficient	not efficient
Scenario 47	50-75-15-9-NELSON-VDT600-none	68.37	10.54	32,825	314,731	44,293	22,752	13.41	28,917,092,962	7,016,858,689	22,295	213,766	not efficient	not efficient
Scenario 56	50-75-15-9-NLST-GR12.5-none	68.06	10.95	34,122	325,575	42,717	28,483	15.84	29,748,996,225	7,848,761,952	24,107	230,021	not efficient	not efficient
Scenario 54	50-75-15-9-NLST-GR10-none	68.40	11.08	34,525	329,167	42,610	29,643	16.29	29,830,458,906	7,930,224,633	24,092	229,695	not efficient	not efficient
Scenario 55	50-75-15-9-NLST-GR7.5-none	68.64	11.16	34,757	331,214	42,520	30,791	16.76	29,902,320,995	8,002,086,722	24,160	230,229	not efficient	not efficient
Scenario 57	50-75-15-9-NLST- Dfup5	68.05	10.83	33,738	333,574	44,213	29,610	17.04	29,682,843,414	7,782,609,141	23,331	230,678	not efficient	not efficient
Scenario 48	50-75-15-9-NELSON-Vfup80	69.39	11.02	34,317	337,355	44,808	29,451	17.05	29,191,246,090	7,291,011,817	21,612	212,461	not efficient	not efficient
Scenario 53	50-75-15-9-NLST-GR12.5-D10	67.49	11.05	34,418	339,475	42,368	30,993	17.17	29,828,497,218	7,928,262,945	23,355	230,352	not efficient	not efficient
Scenario 51	50-75-15-9-NLST-GR10-D11	67.69	11.13	34,673	340,726	42,383	31,253	17.24	29,854,521,936	7,954,287,663	23,345	229,409	not efficient	not efficient
Scenario 49	50-75-15-9-NLST- GR10-D10	68.54	11.30	35,193	346,100	42,333	31,385	17.30	29,885,787,583	7,985,553,310	23,073	226,907	not efficient	not efficient
Scenario 40	50-75-15-9-NELSON-VDT400-V750	69.77	11.43	35,603	347,754	43,379	29,951	16.83	29,273,891,072	7,373,656,799	21,204	207,108	not efficient	not efficient
Scenario 52	50-75-15-9-NLST-GR7.5-D10	69.17	11.44	35,622	349,465	42,322	31,706	17.41	29,926,417,893	8,026,183,620	22,967	225,315	not efficient	not efficient
Scenario 50	50-75-15-9-NLST-GR10-D9	69.37	11.45	35,669	350,891	42,301	31,560	17.37	29,918,079,355	8,017,845,082	22,850	224,785	not efficient	not efficient
Scenario 43	50-75-15-9-NELSON-VDT300-V500	71.00	11.67	36,352	355,128	43,349	30,891	17.22	29,351,505,809	7,451,271,536	20,982	204,976	not efficient	not efficient
Scenario 39	50-75-15-9-NELSON-VDT400-V500	71.10	11.68	36,387	355,348	43,343	31,005	17.27	29,363,651,302	7,463,417,029	21,003	205,112	not efficient	not efficient
Scenario 44	50-75-15-9-NELSON-VDT600-V500	71.30	11.71	36,465	355,742	43,335	31,770	17.60	29,408,338,707	7,508,104,434	21,105	205,899	not efficient	not efficient
Scenario 42	50-75-15-9-NELSON-VDT400-V400	71.72	11.78	36,709	358,394	43,334	31,596	17.52	29,409,436,683	7,509,202,410	20,952	204,560	not efficient	not efficient
Scenario 41	50-75-15-9-NELSON-VDT400-V300	72.39	11.90	37,075	362,039	43,331	32,183	17.78	29,455,834,679	7,555,600,405	20,870	203,792	23,837	285,630

The scenarios are sorted arranging life years gained in ascending order.

2.4. Cost-effectiveness of the efficient screening scenarios in the sensitivity analyses

Table S13: Cost-effectiveness of the efficient screening scenarios in the sensitivity analyses.

Sensitivity analysis assumption	Scenario characteristics	Detected cancers at an early stage (I/II), %	Reduction in lung cancer mortality, %	Lung cancer deaths averted	Discounted life years gained	Interval cancer cases	Over diagnosed cases	Over diagnosis, %	Discounted total cost, million Euro	Discounted additional costs vs no screening, million Euro	Cost per life years gained vs no screening (uniform discounting) Euro	Discounted cost per lung cancer death averted vs no screening, Euro
**Decreased adherence (85%)**	55-75-40-10-NELSON-VDT300-only	67.88	11.04	15,951	147,905	18,266	14,241	17.51	11,128,381,492	2,468,209,650	16,687.80	154,736.99
	55-75-40-10-NELSON-VDT400-V300	70.48	11.71	16,926	154,633	17,939	17,447	19.99	11,327,266,138	2,667,094,296	17,247.89	157,573.81
	50-75-15-9-NELSON-VDT400-V300	71.59	11.20	34,885	339,158	39,059	31,360	18.12	28,876,917,490	6,976,683,217	20,570.59	199,990.92
**Decreased CT sensitivity**	55-75-40-10-NELSON-VDT300-only	65.91	8.51	12,290	113,926	28,728	5,822	9.40	10,769,522,882	2,109,351,040	18,515.04	171,631.49
	55-75-40-10-NELSON-VDT400-V300	69.81	10.75	15,538	142,709	25,783	16,697	20.53	11,362,508,546	2,702,336,704	18,935.96	173,917.92
	50-75-15-9-NELSON-VDT400-V300	70.76	10.31	32,114	312,327	55,624	30,160	18.61	29,154,812,658	7,254,578,384	23,227.53	225,900.80
**Increased CT sensitivity**	55-75-40-10-NELSON-VDT300-only	68.97	10.42	15,061	139,346	22,429	7,189	9.82	10,946,629,549	2,286,457,708	16,408.45	151,813.14
	55-75-40-10-NELSON-VDT400-V300	72.33	12.81	18,507	170,216	19,258	18,347	19.72	11,566,774,883	2,906,603,041	17,075.97	157,054.25
	50-75-15-9-NELSON-VDT400-V300	73.41	12.34	38,450	375,036	41,991	33,020	17.80	29,561,115,978	7,660,881,704	20,427.06	199,242.70
**Survival 20%**	55-75-40-10-NELSON-VDT300-only	67.31	4.90	7,073	64,976	23,057	6,733	9.48	10,892,118,388	2,231,946,546	34,350.47	315,558.68
	55-75-40-10-NELSON-VDT400-V300	71.35	6.15	8,886	81,476	19,854	17,892	19.69	11,515,704,975	2,855,533,133	35,047.54	321,351.92
	50-75-15-9-NELSON-VDT400-V300	72.39	5.90	18,383	179,031	43,331	32,183	17.78	29,455,834,680	7,555,600,406	42,202.87	411,010.19
**Survival 30%**	55-75-40-10-NELSON-VDT300-only	67.31	7.39	10,671	98,141	23,057	6,733	9.48	10,892,118,388	2,231,946,546	22,742.13	209,160.02
	55-75-40-10-NELSON-VDT400-V300	71.35	9.25	13,372	122,191	19,854	17,892	19.69	11,515,704,975	2,855,533,133	23,369.47	213,545.70
	50-75-15-9-NELSON-VDT400-V300	72.39	8.85	27,577	268,594	43,331	32,183	17.78	29,455,834,680	7,555,600,406	28,130.18	273,981.96
**Survival 50%**	55-75-40-10-NELSON-VDT300-only	67.31	12.31	17,788	163,572	23,057	6,733	9.48	10,892,118,388	2,231,946,546	13,645.04	125,474.85
	55-75-40-10-NELSON-VDT400-V300	71.35	15.38	22,218	203,495	19,854	17,892	19.69	11,515,704,975	2,855,533,133	14,032.47	128,523.41
	50-75-15-9-NELSON-VDT400-V300	72.39	14.76	45,972	448,474	43,331	32,183	17.78	29,455,834,680	7,555,600,406	16,847.36	164,352.22
**Survival 60%**	55-75-40-10-NELSON-VDT300-only	67.31	17.34	21,349	196,872	23,057	6,733	9.48	10,892,118,388	2,231,946,546	11,337.03	104,545.72
	55-75-40-10-NELSON-VDT400-V300	71.35	22.64	26,676	245,212	19,854	17,892	19.69	11,515,704,975	2,855,533,133	11,645.18	107,045.03
	50-75-15-9-NELSON-VDT400-V300	72.39	21.69	55,518	541,052	43,331	32,183	17.78	29,455,834,680	7,555,600,406	13,964.66	136,092.81
**Cost per CT 200 Euro**	55-75-40-10-NELSON-VDT300-only	67.31	9.95	14,373	133,222	23,057	6,733	9.48	11,348,079,792	2,687,907,951	20,176.16	187,010.92
	55-75-40-10-NELSON-VDT400-V300	71.35	12.38	17,889	164,864	19,854	17,892	19.69	11,971,666,380	3,311,494,538	20,086.20	185,113.45
	50-75-15-9-NELSON-VDT400-V300	72.39	11.90	37,075	362,039	43,331	32,183	17.78	31,003,818,424	9,103,584,150	25,145.34	245,545.09
**Cost per CT 500 Euro**	55-75-40-10-NELSON-VDT300-only	67.31	9.95	14,373	133,222	23,057	6,733	9.48	14,083,848,222	5,423,676,380	40,711.57	377,351.73
	55-75-40-10-NELSON-VDT400-V300	71.35	12.38	17,889	164,864	19,854	17,892	19.69	14,707,434,810	6,047,262,968	36,680.28	338,043.66
	50-75-15-9-NELSON-VDT400-V300	72.39	11.90	37,075	362,039	43,331	32,183	17.78	40,291,720,874	18,391,486,601	50,799.80	496,061.68
**Cost per CT 100 Euro**	55-75-40-10-NELSON-VDT300-only	67.31	9.95	14,373	133,222	23,057	6,733	9.48	10,436,156,983	1,775,985,141	13,331.02	123,563.98
	55-75-40-10-NELSON-VDT400-V300	71.35	12.38	17,889	164,864	19,854	17,892	19.69	11,059,743,569	2,399,571,727	14,554.84	134,136.72
	50-75-15-9-NELSON-VDT400-V300	72.39	11.90	37,075	362,039	43,331	32,183	17.78	27,907,850,938	6,007,616,665	16,593.86	162,039.56
**Innovative Treatment Cost**	55-75-40-10-NELSON-VDT300-only	67.31	9.95	14,373	133,222	23,057	6,733	9.48	25,486,357,786	3,539,339,140	26,567.23	246,249.16
	55-75-40-10-NELSON-VDT400-V300	71.35	12.38	17,889	164,864	19,854	17,892	19.69	27,055,830,817	5,108,812,172	30,988.01	285,584.00
	50-75-15-9-NELSON-VDT400-V300	72.39	11.90	37,075	362,039	43,331	32,183	17.78	66,668,886,830	11,873,009,409	32,794.87	320,243.00

Table S 14: Comparison of the microsimulation model outcomes with the data from the NLST trial.

	NLST	55-74-30-15-NLST-GR10-D10
Follow-up after end of annual screening	median 6.5 years	7 years*
Screen exams per person	2.8	3.8
Lung Cancer specific mortality rate per 100,000 person-years:		
LDCT	247	332
Radiography/no screening	309	394
Difference in mortality rates	62	62
Lung cancer mortality reduction, %	20.1	15.8
All-cause mortality rate per 100,000 person-years		
LDCT	1,303	1,930
Radiography/no screening	1,395	1,986
Mortality reduction absolute	92	56
Screen detected Lung Cancer:		
Proportion of all detected cancer, %	61.2	67.9
Stage I, %	63.0	59.6
Stage II, %	7.2	8.3
Stage III, %	17.0	19.5
Stage IV, %	12.8	12.6
Small-cell carcinoma, %	7.6	5.4
Large-cell carcinoma, %	4.3	2.8
Squamous-cell carcinoma, %	21.1	23.2
Adenocarcinoma, %	39.9	50.0
Adenocarcinoma *in situ*, %	14.7	18.6
Non-small-cell carcinoma or other, %	11.6	n/a
Carcinoid, %	0.8	n/a

* for comparison purposes. The model simulates a follow-up over a lifetime course.

References

1 Scheidt-Nave C, Du Y, Knopf H, et al. Verbreitung von Fettstoffwechselstörungen bei Erwachsenen in Deutschland. *Bundesgesundheitsbl.* 2013; **56:** 661–67. doi:10.1007/s00103-013-1670-0.

2 Statistisches Bundesamt. Ergebnisse auf Grundlage des Zensus 2011. Wiesbaden, 2016.

3 Woloshin S, Schwartz LM, Welch HG. The risk of death by age, sex, and smoking status in the United States: putting health risks in context. *Journal of the National Cancer Institute* 2008; **100:** 845–53. doi:10.1093/jnci/djn124.

4 Moolgavkar SH, Luebeck G. Two-Event Model for Carcinogenesis: Biological, Mathematical, and Statistical Considerations. *Risk Analysis* 1990; **10:** 323–41. doi:10.1111/j.1539-6924.1990.tb01053.x.

5 McMahon PM, Kong CY, Johnson BE, et al. Chapter 9: The MGH-HMS Lung Cancer Policy Model: Tobacco Control Versus Screening. *Risk Analysis* 2012; **32:** S117-S124. doi:10.1111/j.1539-6924.2011.01652.x.

6 Lung Cancer: Current Diagnosis and Treatment.

7 Erasmus University Medical Center. MISCAN-Lung (Erasmus) MIcrosimulation SCreening Analysis (MISCAN) Lung Model. CISNET_ModelProfile_LUNG_ERASMUS_001_01132012_83607.pdf (accessed Jul 28, 2016).

8 Chrysanthopoulou AS. MIcrosimulation Lung Cancer (MILC) model, Package ‘MILC’. https://cran.r-project.org/web/packages/MILC/MILC.pdf.

9 Hazelton WD, Clements MS, Moolgavkar SH. Multistage carcinogenesis and lung cancer mortality in three cohorts. *Cancer epidemiology, biomarkers & prevention : a publication of the American Association for Cancer Research, cosponsored by the American Society of Preventive Oncology* 2005; **14:** 1171–81. doi:10.1158/1055-9965.EPI-04-0756.

10 Zentrum für Krebsregister. Lungenkrebs (Bronchialkarzinom). http://www.krebsdaten.de/Krebs/DE/Content/Krebsarten/Lungenkrebs/lungenkrebs_node.html (accessed May 12, 2016).

11 Eberle A, Jansen L, Castro F, et al. Lung cancer survival in Germany: A population-based analysis of 132,612 lung cancer patients. *Lung Cancer* 2015; **90:** 528–33. doi:10.1016/j.lungcan.2015.10.007.

12 Horeweg N, Scholten ET, de Jong, Pim A, et al. Detection of lung cancer through low-dose CT screening (NELSON): a prespecified analysis of screening test performance and interval cancers. *The Lancet Oncology* 2014; **15:** 1342–50. doi:10.1016/S1470-2045(14)70387-0.

13 Aberle DR, Adams AM, Berg CD, et al. Reduced lung-cancer mortality with low-dose computed tomographic screening. *The New England journal of medicine* 2011; **365:** 395–409. doi:10.1056/NEJMoa1102873.

14 Kvale PA, Johnson CC, Tammemagi M, et al. Interval lung cancers not detected on screening chest X-rays: How are they different? *Lung cancer (Amsterdam, Netherlands)* 2014; **86:** 41–46. doi:10.1016/j.lungcan.2014.07.013.

15 Bach PB, Mirkin JN, Oliver TK, et al. Benefits and harms of CT screening for lung cancer: a systematic review. *JAMA* 2012; **307:** 2418–29. doi:10.1001/jama.2012.5521.

16 McMahon PM, Kong CY, Bouzan C, et al. Cost-Effectiveness of Computed Tomography Screening for Lung Cancer in the United States. *Journal of Thoracic Oncology* 2011; **6:** 1841–48. doi:10.1097/JTO.0b013e31822e59b3.

17 Yeow K-M, Su I-H, Pan K-T, et al. Risk Factors of Pneumothorax and Bleeding. *Chest* 2004; **126:** 748–54. doi:10.1378/chest.126.3.748.

18 Soetaert K, Petzoldt T. Inverse Modelling, Sensitivity and Monte Carlo Analysis in R Using Package FME. *J. Stat. Soft.* 2010; **33. doi:**10.18637/jss.v033.i03.

19 Diederich S, Wormanns D, Semik M, et al. Screening for Early Lung Cancer with Low-Dose Spiral CT: Prevalence in 817 Asymptomatic Smokers1. *Radiology* 2002; **222:** 773–81. doi:10.1148/radiol.2223010490.

20 National Association of Statutory Health Insurance Physicians. Uniform Value Scale. http://www.kbv.de/html/ebm.php (accessed Jul 27, 2016).

21 German Institute for the Hospital Remuneration System (InEK). G-DRG catalog. http://www.g-drg.de (accessed Jul 27, 2016).

22 Geraghty PR, Kee ST, McFarlane G, Razavi MK, Sze DY, Dake MD. CT-guided Transthoracic Needle Aspiration Biopsy of Pulmonary Nodules: Needle Size and Pneumothorax Rate1. *Radiology* 2003; **229:** 475–81. doi:10.1148/radiol.2291020499.

23 McGuire A, Martin M, Lenz C, Sollano JA. Treatment cost of non-small cell lung cancer in three European countries: comparisons across France, Germany, and England using administrative databases. *Journal of medical economics* 2015; **18:** 525–32. doi:10.3111/13696998.2015.1032974.

24 Department of Health. The likely impact of earlier diagnosis of cancer on costs and benefits to the NHS. http://www.dh.gov.uk/publications (accessed Aug 17, 2016).

25 Buckland D. New drug treatments for cancer. What the future holds. *Prescriber* 2016; **27:** 17–21. doi:10.1002/psb.1425.
26 Schremser K, Rogowski WH, Adler-Reichel S, Tufman ALH, Huber RM, Stollenwerk B. Cost-Effectiveness of an Individualized First-Line Treatment Strategy Offering Erlotinib Based on EGFR Mutation Testing in Advanced Lung Adenocarcinoma Patients in Germany. *PharmacoEconomics* 2015; **33:** 1215–28. doi:10.1007/s40273-015-0305-8.
